# The EDEN ISS mobile test facility microbiome changes by cleaning and continued use

**DOI:** 10.3389/frmbi.2025.1608732

**Published:** 2025-10-17

**Authors:** Luis Gaiser, Kristina Beblo-Vranesevic, Rob Van Houdt, Jana Fahrion, Louise Gillet de Chalonge, Jess M. Bunchek, Paul Zabel, Daniel Schubert, Petra Rettberg

**Affiliations:** ^1^ Aerospace Microbiology Working Group, Department of Applied Aerospace Biology, Institute for Aerospace Medicine, German Aerospace Center (DLR), Cologne, Germany; ^2^ Microbiology Unit, Belgian Nuclear Research Centre (SCK CEN), Mol, Belgium; ^3^ Institut Pascal, Clermont Auvergne INP, CNRS, Université Clermont Auvergne, Clermont-Ferrand, France; ^4^ Life Sciences Institute, School of Chemical Science, Dublin City University, Dublin, Ireland; ^5^ Planetary Infrastructures Working Group, Department of System Analysis Space Segment, Institute of Space Systems, German Aerospace Center (DLR), Bremen, Germany

**Keywords:** bioregenerative life support system (BLSS), greenhouse, surface microbiome, plant cultivation technologies, Antarctica, built environment

## Abstract

**Introduction:**

Bioregenerative life support systems (BLSS) utilizing plants and/or microorganisms to provide the crew of a spacecraft with food, clean water, breathable air, and other amenities are likely to form key components of future long-distance spaceflight missions. Extensive testing and validation of such technologies are necessary before they can be implemented. EDEN ISS was a platform in Antarctica that tested various plant cultivation technologies for a BLSS. To ensure the continued operation of a BLSS, it is vital that plants remain healthy, which necessitates the monitoring of the plant production facility microbiome to ensure that pathogens are detected early and countermeasures can be engaged.

**Methods:**

Swab surface samples collected in the EDEN ISS Mobile Test Facility (MTF) during different campaigns were used to estimate the bioburden of the various surfaces via viable count. Isolates obtained from the cultivation of the surface samples were identified via partial 16S rRNA gene sequencing. Additionally, 16S amplicon sequencing was performed on DNA extracted directly from the swab samples to characterize the microbiome.

**Results and discussion:**

The results revealed that the bioburden of the different sampling positions was not significantly reduced by cleaning, indicating that the employed cleaning regime was unsuited in its current form to adequately lower the bioburden. Identification of the isolates, as well as the full microbiome, revealed mostly environmental genera. However, in both cases, genera containing plant as well as human pathogens, like *Pseudomonas* and *Acinetobacter*, were identified and accounted for up to 16.1% of all reads for a sampling condition in the case of *Pseudomonas*. The two sets of sequencing data had little overlap, with *Rhodococcus* and *Microbacterium* being the only genera shared between all sampling conditions and sequencing approaches, and emphasized different aspects of the MTF microbiome, highlighting the advantages of using a combined approach to obtain a more complete picture of the microbiome composition.

## 1 Introduction

The biggest limitations of crewed spaceflight regarding duration and distance, besides the space environment itself (i.e., radiation and low gravity), are the mass and storage requirements for pre-packaged supplies like food, water, and medicine. For example, a mission to Mars would require stocking more than one ton of food per crew member (Pickett et al., 2020), quickly reaching the limits of storage space and propellant. A solution for these challenges is developing technologies that allow for the regeneration of all needed resources from a small initial supply. One approach to realize this is to employ the regenerative capabilities found in nature by utilizing plants, microbes, and other organisms for recycling resources in bioregenerative life support systems (BLSS). Once sufficiently developed, a BLSS could provide a crew with breathable atmosphere, clean water, and food and even enable the *in situ* production of pharmaceuticals and other natural products, all while forming an almost completely closed loop (Liu et al., 2021).

Higher plants have been shown to fulfill a large range of these needs because of their ability to perform photosynthesis. This makes plants attractive targets for the development of BLSS technologies (Mitchell, 1994; Gòdia et al., 2002) by consuming the CO_2_ produced by the crew and providing O_2_ as a byproduct (Singhal, 2012). For instance, during the Lunar Palace 1 experiment, a growing area of 69 m² was sufficient to provide breathable air for three crewmembers over a duration of 105 days (Liu et al., 2021). Some plants can also play a valuable role in water reclamation, as plant xylem possesses pores ideally sized to filter out pathogens and small contaminants (Boutilier et al., 2014). This allows for the irrigation of some species of plants with pretreated wastewater and reclaiming the purified water from the water vapor released from the plant stomata, although this might make the plants unsafe for consumption. Such beneficial functions can also be carried out by microorganisms (De Micco et al., 2023). For example, cyanobacteria can be utilized in air revitalization, thanks to their photosynthetic activity (Keller et al., 2023), while *Nitrosomonas* and *Nitrobacter* species can aid in wastewater reclamation through the removal of nitrogen (Daims et al., 2006). Plants also possess many advantages in regard to food production. They provide a regenerative source of nutrients that can be produced from seeds, which are very storage-, space-, and weight-efficient (Barta and Henninger, 1994). Plants can provide the crew with a balanced and varied diet that meets the majority of their nutritional needs without an overreliance on pre-packaged supplements (Creuly et al., 2005). In principle, food production from non-phototrophic microbial sources would also be possible (Bell et al., 2022); however, plants provide significant advantages for the mental wellbeing of the crew that microbial food sources cannot match. Food produced by microbes could reinforce feelings of homesickness and isolation due to their unfamiliar appearance, taste, or texture and thus negatively influence the mental wellbeing of the crew (Douglas et al., 2020). Caring for plants, on the other hand, provides a connection to Earth through familiar sights, smells, and taste. This helps reduce the negative emotions experienced by the crew (Hirsch, 2013).

Before BLSS can be incorporated into an actual space mission, all of its subcomponents, including plant cultivation, need to be extensively tested. An unexpected failure of the system, for example, through plant diseases caused by pathogens, could be life-threatening for the crew. Testing in an actual space context would be optimal, as it possesses the major advantage of the tests being done in the environment for which the systems are being developed. However, such experiments are constrained in terms of costs, limited scope due to low amounts of available space, and a complicated execution (Nguyen et al., 2023). For these reasons, testing plant cultivation technologies in space analog environments is becoming increasingly relevant. Antarctica presents itself as an ideal environment for such tests, as its harsh climate makes it nearly as inhospitable as space itself. Additionally, the crews of Antarctic research stations are faced with extended periods of isolation, similar to that experienced during crewed space flight, and they have a similar dependence on external resources for survival.

Over the years, multiple, different experimental plant production facilities have been set up in Antarctica (Patterson et al., 2011; Panova et al., 2023), including EDEN ISS. This European Commission project was an international cooperation led by the German Aerospace Center (DLR), with partners from the European Union (EU), the United States (US), and Canada. Its goal lies in the development and experimental validation of plant cultivation systems in a BLSS context (Zabel et al., 2015). It sets itself apart from the earlier plant production facilities by being a standalone facility instead of being contained inside a larger station.

The core component of EDEN ISS was the MTF, which was constructed of two connected shipping containers and included all of the technology required for plant cultivation in remote locations like Antarctica. It was installed in 2017, approximately 400 m away from the German Antarctic research station, Neumayer Station III (NM-III). The MTF started operating in January of 2018 and was in operation during four overwintering expeditions until February 2022. Plant cultivation was carried out in plant growth trays stored in racks, providing for a total plant growth area of 12.5 m² (Zabel et al., 2020). The plants received nutrients via aeroponics. Lighting for the plants was provided by LED lamps with a mixture of blue, red, green, and white light, with individually adjustable outputs and lighting schedules depending on the specific needs of the plants. The environmental conditions in the MTF were controlled by the atmosphere management system (AMS). It replaced the warmer, more humid, and O_2_-richer air produced by the plants through gas exchange with drier, colder, and CO_2_-richer air (Zabel et al., 2017). Cameras that monitored the plants were used for early detection of diseases and damage to the plants (Zeidler et al., 2019). These systems were used successfully to provide the NM-III crew with a wide variety of fruits and vegetables, including cucumbers, tomatoes, peppers, leafy greens, and herbs. Close to 270 kg of edible biomass was produced during the first growing period, which started in January 2018 and lasted until November 2018 (Zabel et al., 2020), and nearly 316 kg was produced in the final growing period, which started in March 2021 and lasted until February 2022 (Vrakking et al., 2022). Access to fresh fruits and vegetables was reported to have had a positive psychological impact on the crew members, as fresh produce was not available during the isolation phase at NM-III (Schlacht et al., 2019).

The health of cultivated plants is vitally important in a BLSS, as plant death, for example, caused by pathogen-induced diseases, impairs recycling of the breathable atmosphere, posing a severe risk to crew survival. Diseases can also impact the production of edible biomass significantly, leading to food scarcity. Premature plant death can also result in a lack of viable seeds, limiting the number of plants that can be produced in the next planting cycle. Additionally, sections of a plant production system or the plants themselves might also serve as breeding grounds for human pathogens (Fletcher et al., 2013; Taormina et al., 1999). These potential health concerns highlight the need for monitoring the microbiome in plant cultivation test facilities like EDEN ISS and evaluating cleaning regimens to remove potentially harmful microbes. Many studies exist that examine the microbiome of surfaces on the International Space Station (ISS) (Ichijo et al., 2020; Checinska Sielaff et al., 2019) and other enclosed environments used to simulate spaceflight conditions, like Mars500 (Schwendner et al., 2017). There have also been various systems to study plant cultivation on the ISS (Kittang et al., 2014; Massa et al., 2017). However, these microbiome monitoring efforts have thus far not included the plant cultivation systems in a comprehensive manner. This work attempts to start filling the knowledge gaps that exist in regard to the microbiome composition of plant cultivation systems for space applications.

A standard approach to determine the amount of viable microbes found in an environment is microbe extraction from surface swabs (Jansson et al., 2020), liquid samples (Obire et al., 2009), or air filters (Torloni and Borzani, 1958), followed by cultivation and enumeration by counting or photometric measurements (Yusof, 2001). This approach is suitable to evaluate the degree to which an environment is colonized by microorganisms, informs about the effectiveness of an applied cleaning method (White et al., 2007), and is used to validate the decontamination of clean rooms in a standardized way (Mora et al., 2016). However, additional steps are necessary to identify the cultivated organisms. Due to the time- and labor-intensive nature and limitations in identifying species via classic culture-based approaches (Petti et al., 2005), culture-independent approaches have been developed. The sequencing of the 16S rRNA gene for bacteria and the internal transcribed spacer (ITS) sequences for fungi are the gold standards (Baldwin et al., 1995; Buszewski et al., 2019).

Although sequencing DNA obtained from cultivated isolates ensures that the identified organisms were viable in the environment when sampled, this approach does not capture the wide variety of all species present, as it is estimated that only 1% of all species are currently able to be cultivated (Staley and Konopka, 1985). Species that are able to grow better under the chosen culture conditions might also be overrepresented and not accurately reflect their actual abundance in the sampled environment (Hugenholtz, 2002). Sequencing DNA directly from surface swabs, wipes, or air filters has the advantage of more accurately reflecting the variety of species present in the sampled environment; however, it is unable to differentiate whether it originates from a viable organism, a dead cell, or even free DNA (Hugenholtz, 2002). Applying a combination of culture-dependent and culture-independent methods will result in a more complete picture of the microbiome.

The goal of this work was to analyze various surface samples obtained from the EDEN ISS MTF to understand its microbiome composition, emphasizing the identification of pathogens. Additionally, combining sequencing- and culture-based approaches to gather a more complete picture of the true microbiome composition was evaluated.

## 2 Material and methods

### 2.1 Sampling in the EDEN ISS MTF

**Figure 1:**
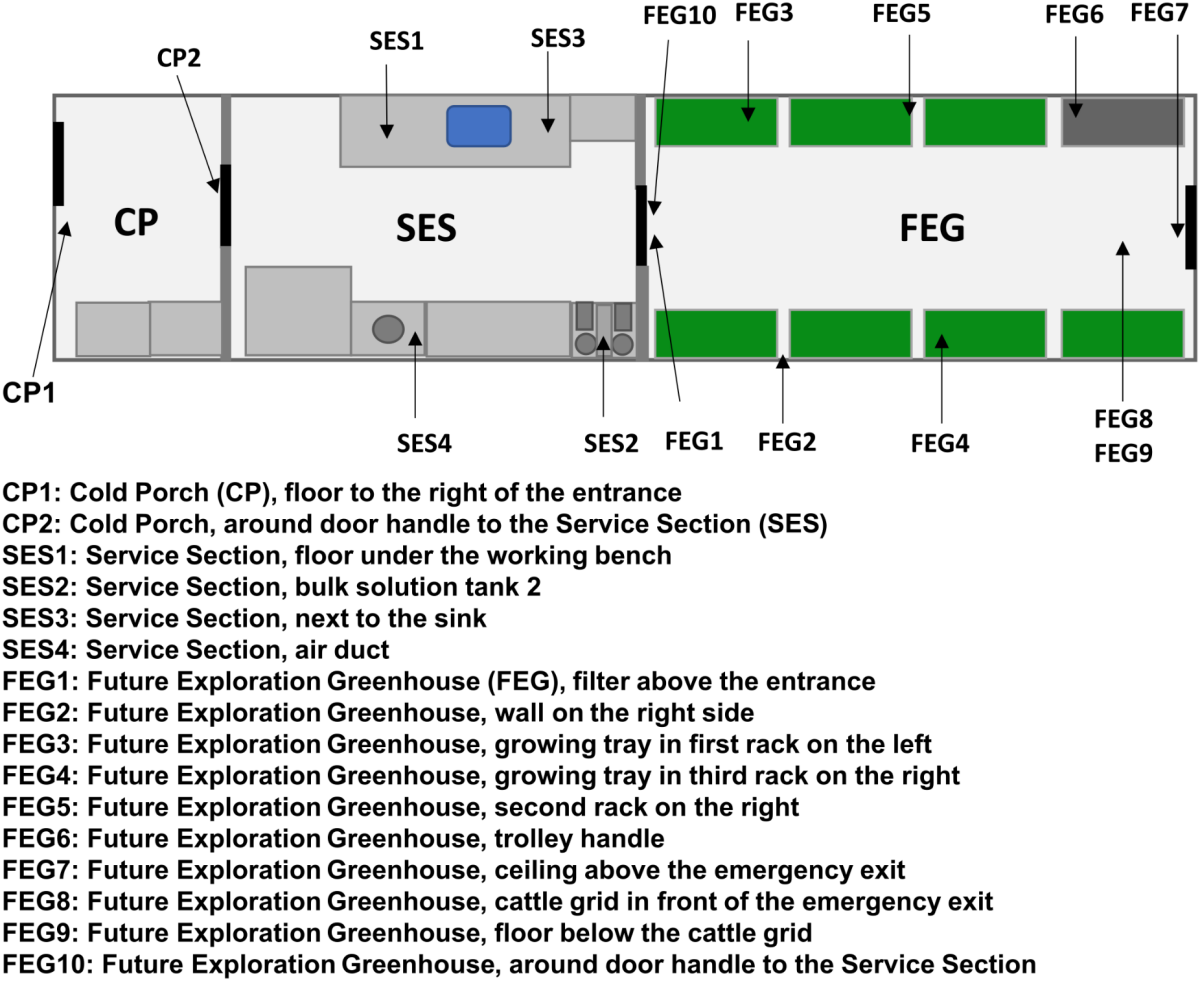
EDEN ISS sampling locations. The schematic view of the EDEN ISS container shows the locations of the sampled positions in the Future Exploration Greenhouse (FEG), the Service Section (SES), and the Cold Porch (CP).

The samples from EDEN ISS were taken during two separate sampling campaigns. The first sampling campaign included two sampling dates in December 2019 and January 2020. The first sampling date, referred to as pre-cleaning, took place before maintenance work, and thorough cleaning was carried out in the MTF between two growing seasons. The maintenance work and cleaning were carried out over the duration of 1 month. Growing trays and other movable components were cleaned and stored at the station at the beginning, while the surfaces were cleaned after the maintenance work was completed. The second sampling date, referred to as post-cleaning, took place 4 weeks later after this work had finished. The sampling locations (**Figure 1**) included various surfaces inside the MTF, like door handles, the floor and walls, and plant-growing trays, and were identical to the ones monitored in a previous study of the MTF microbiome (Fahrion et al., 2020), from which most of the sample preparation methods were adapted.

The second sampling campaign took place during the growing season from March 2021 to January 2022. The sampling locations in this campaign were limited to one wall of the MTF (FEG2) and the floor beneath the cattle grid (FEG9), as these two locations were identified to possess a high bioburden during the previous study. Samples were taken once a week for 16 weeks, after which the sampling was carried out monthly for another 7 months.

Surface samples for cultivation were obtained by wetting a sterile FlOQSwab™ (Copan, Brescia, Italy) with sterile deionized water, wiping down an area of 25 cm² (5 cm × 5 cm) and depositing the swab head in a 15-mL reaction tube (Falcon) containing 2.5 mL of sterile phosphate buffered saline (PBS, 10 g of NaCl, 0.25 g of KCl, 2.26 g of Na_2_HPO_4_, and 0.3 g of KH_2_PO_4_ per liter of Millipore water). Negative controls were obtained by taking a sterile swab, waving it in the air of the respective compartment of the MTF for a few seconds, and then depositing it in a 15-mL reaction tube in the same way as the samples. All samples and controls were taken in duplicates.

Additionally, surface swabs for DNA extraction were taken. The sampling was carried out in an identical fashion to the cultivation samples; however, these swabs were stored dry in the tube of the swab without any liquid. All of these samples were taken in duplicates. The samples were stored and shipped at −40°C in a temperature-controlled container for further analysis. At the analysis site, the samples were stored at −70°C until they were processed.

### 2.2 Cleaning of the MTF

Cleaning of the MTF was carried out as follows: during the first sampling campaign, which took place between growing seasons, so no plants were present at the time, all equipment was disassembled for maintenance before the MTF was cleaned. The growing trays were taken into NM-III to be cleaned. The growing trays and working surfaces were cleaned with hot water and a commercial dish soap (Pril Original, Henkel, Germany) and wiped down with 90%–95% ethanol. The floor, walls, and ceiling were wiped with a wet mop. The air filters, which were made from a tight wire mesh, were exchanged with new ones. Before the MTF was put back into operation, a nebulizer was used to mist the entire facility with 30% H_2_O_2_ for approximately 90 min to sterilize the surfaces. During cleaning, the temperature levels in the MTF were comparable to the levels during normal operation of 19°C to 21°C, while the humidity was lower than the setpoint of 65% during normal operation (Zabel et al., 2020). During the second campaign, when the MTF was in operation, the subfloor, where FEG9 was located, had to be periodically cleaned. This was done when visible mold growth or biofilm formation was observed. While cleaning, the debris was removed with a wet sponge, taking care not to disturb the FEG9 sampling area. Mold and biofilms not in the vicinity of the sampling site were removed by treating the contaminated area with 0.5 M of H_2_O_2_, while the sampling area was cleaned using a vacuum and dry paper towels. Condensation on the subfloor was removed by vacuuming.

### 2.3 Sample preparation from surface swabs

The swabs stored frozen in liquid were thawed for 2 h at room temperature. The swabs were vortexed for approximately 10 s and subsequently sonicated for 2 min at 40 kHz to remove as many cells as possible. Five hundred microliters of the suspension was pipetted into a 1.5-mL microcentrifuge tube (Eppendorf, Hamburg, Germany) for a heat shock at 80°C for 15 min, while an additional 500 µL was taken to be used without heat shock. The remaining suspension was put into a separate microcentrifuge tube and stored at −20°C as backup.

### 2.4 Sample plating

The untreated and the heat-shocked samples were serially diluted from 10^0^ to 10^−5^ in sterile PBS. Two hundred microliters per plate of each dilution was plated on Reasoner’s 2 agar (R2A) plates containing 50 mg/L of cycloheximide for the cultivation of bacteria while suppressing fungal growth. The plates were incubated at room temperature (~22°C) for 7 days. Subsequently, the number of colonies was counted.

### 2.5 Strain identification

Colonies with unique morphologies from each plate were picked with a sterile inoculation loop and streaked onto a corresponding agar plate to create a collection of cultivated isolates for subsequent identification through sequencing. Purified isolates were identified by partial 16S rRNA gene sequencing. To this end, the 16S rDNA gene was amplified with the universal primers 8F (5′-AGAGTTTGATCCTGGCTCAG-3′) and 1492R (5′-AGAGTTTGATCCTGGCTCAG-3′), which target almost the entire 16S rRNA gene (Weisburg et al., 1991), utilizing the VeriFi^®^ polymerase (PCR Biosystems, Wayne, Pennsylvania, USA) on PrepMan™ Ultra (Thermo Fisher Scientific, Waltham, Massachusetts, USA) treated cells. Purification and sequencing of the PCR products with Sanger sequencing were outsourced (Eurofins, Konstanz, Germany). IDTAXA was used to classify the organisms using the obtained nucleotide sequences (Murali et al., 2018).

### 2.6 DNA extraction from surface swabs

Swabs were thawed for 2 h at room temperature. Both duplicates from the pre- and post-cleaning sampling campaigns were used for extraction but processed separately. After thawing, the swab heads were cut off with a pair of scissors wiped down with Bacillol and transferred into a ZR BashingBead™ Lysis Tube (ZR Group, Irvine, California, USA) containing beads with 0.5 and 0.1 mm in diameter. Subsequently, bead bashing was initiated using a frequency of 30 Hz for 5 min, followed by DNA extraction with the D4301 ZymoBIOMICS DNA Microprep Kit [Zymo Research (ZR) Group, Irvine, California, USA]. The DNA concentration of the final elution was determined with a Qubit fluorometer using the Qubit™ dsDNA-HS (High Sensitivity) Kit (Thermo Fisher Scientific, Waltham, Massachusetts, USA). Afterward, the DNA was stored at −20°C until further use.

### 2.7 16S rRNA next-generation sequencing library preparation

The 16S rRNA gene library was prepared from the extracted DNA with the D6400 Quick-16S™ NGS Library Prep Kit (ZR Group, Irvine, California, USA) according to the manufacturer’s instructions. The Quick-16S™ Primer Set V1-V2 (ZR Group, Irvine, California, USA) was used to amplify the V1-V2 region of the 16S rRNA genes of the DNA sample. The library was sequenced with an Illumina MiSeq utilizing the Illumina MiSeq Reagent Kit v3–2 × 300 bp (Illumina, San Diego, California, USA) resulting in 300-bp amplicons.

### 2.8 Analysis of NGS data

**Figure 2:**
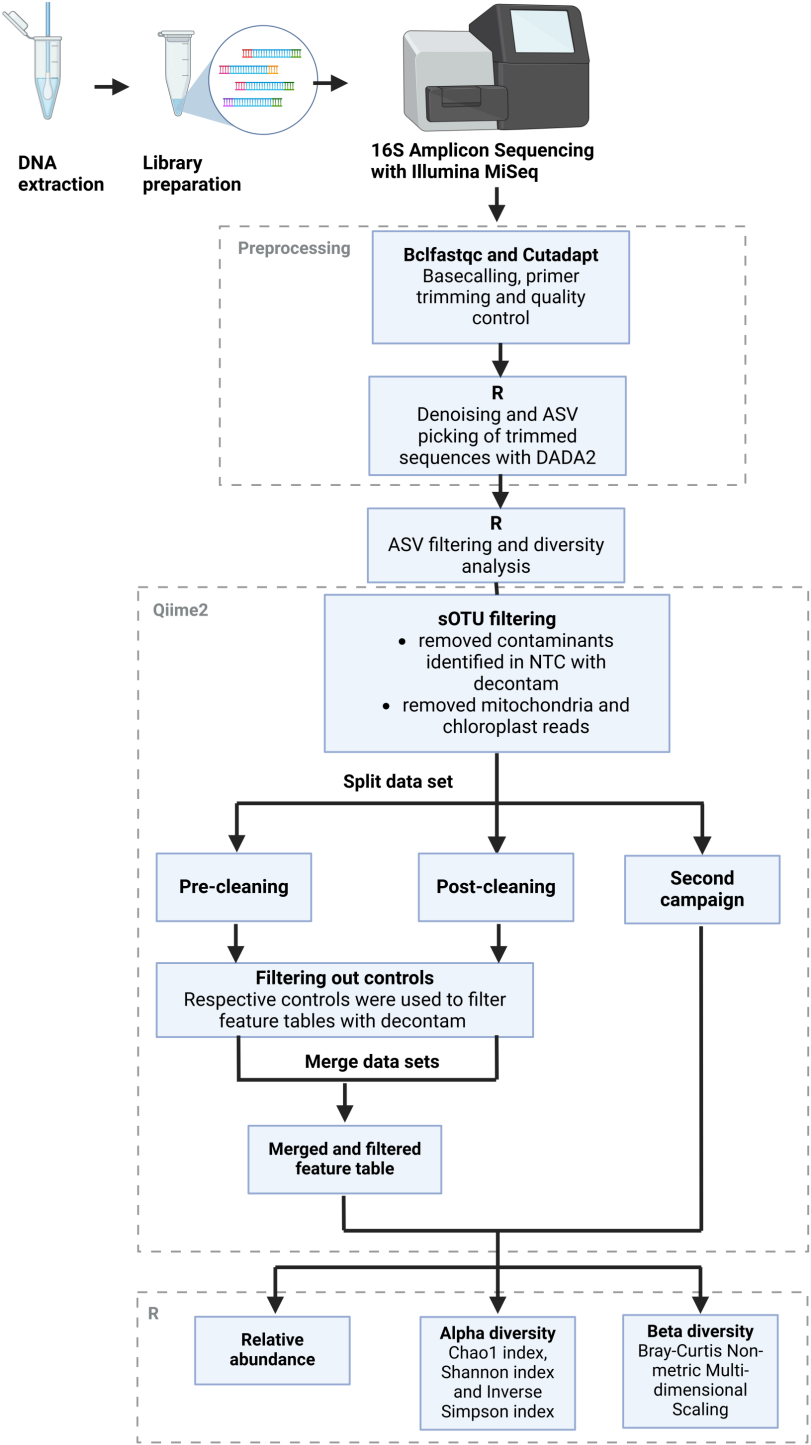
Bioinformatics workflow. Schematic overview of the bioinformatics workflow from the raw reads obtained from Illumina sequencing to the final outputs of relative abundance levels with taxonomic annotations as well as alpha and beta diversity measures. The figure was adapted from Townsend et al. (2023), by modifying the layout to fit this particular workflow. Figure created with Biorender.com.

Bioinformatic analysis of the NGS data was performed largely with the Qiime2 (Bolyen et al., 2018) v. amplicon-2023.9 software in the JupyterHub of the biocomputational facility of the Justus Liebig University Gießen and R version 4.4.2 (**Figure 2**). In a first step, basecalling of the forward and reverse reads from the raw sequences was performed with the “bcl2fastq” package (Illumina, 2024). Afterward, the primer sequences, barcodes, and adapter sequences were trimmed with the “cutadapt” package to a length of 200 bp (Martin, 2011). Quality control of the trimmed sequences was performed with the “fastqc” package (Babraham_Bioinformatics, 2023), and the resulting reports were summarized with the “multiqc” package (Ewels et al., 2016). Sequences with good quality were used for further processing in R. Samples with insufficient or missing results were sequenced again. The datasets from separate sequencing runs were processed in parallel until they could be merged. The DADA2 algorithm (Callahan et al., 2016) was used to group reads into suboperational taxonomic units (ASVs) and assign them a taxonomy with the help of the Silva reference database (Quast et al., 2013). The feature tables and taxonomy files were then exported to Qiime2 to remove mitochondria and chloroplast reads and to identify and remove contaminants with the “decontam” package. The denoising stats are recorded in **Supplementary Table 2**. The filtered feature data were then reimported into R for analysis of the relative abundance levels of common genera as well as alpha and beta diversity analysis using the vegan package (Dixon, 2003). Predictions of the functional potential of the greenhouse microbiome based on its 16S rRNA sequences, especially regarding pathogenic functions, were carried out with the Picrust2 tool (Barbera et al., 2019; Czech et al., 2020; Douglas et al., 2020; Mirarab et al., 2012; Louca and Doebeli, 2018; Ye and Doak, 2009). The resulting output was then annotated with the KEGG BRITE database (Kanehisa et al., 2025; Kanehisa, 2019; Kanehisa and Goto, 2000), using the version from 01 July 2025.

## 3 Results

### 3.1 Bioburden of surface samples

**Figure 3:**
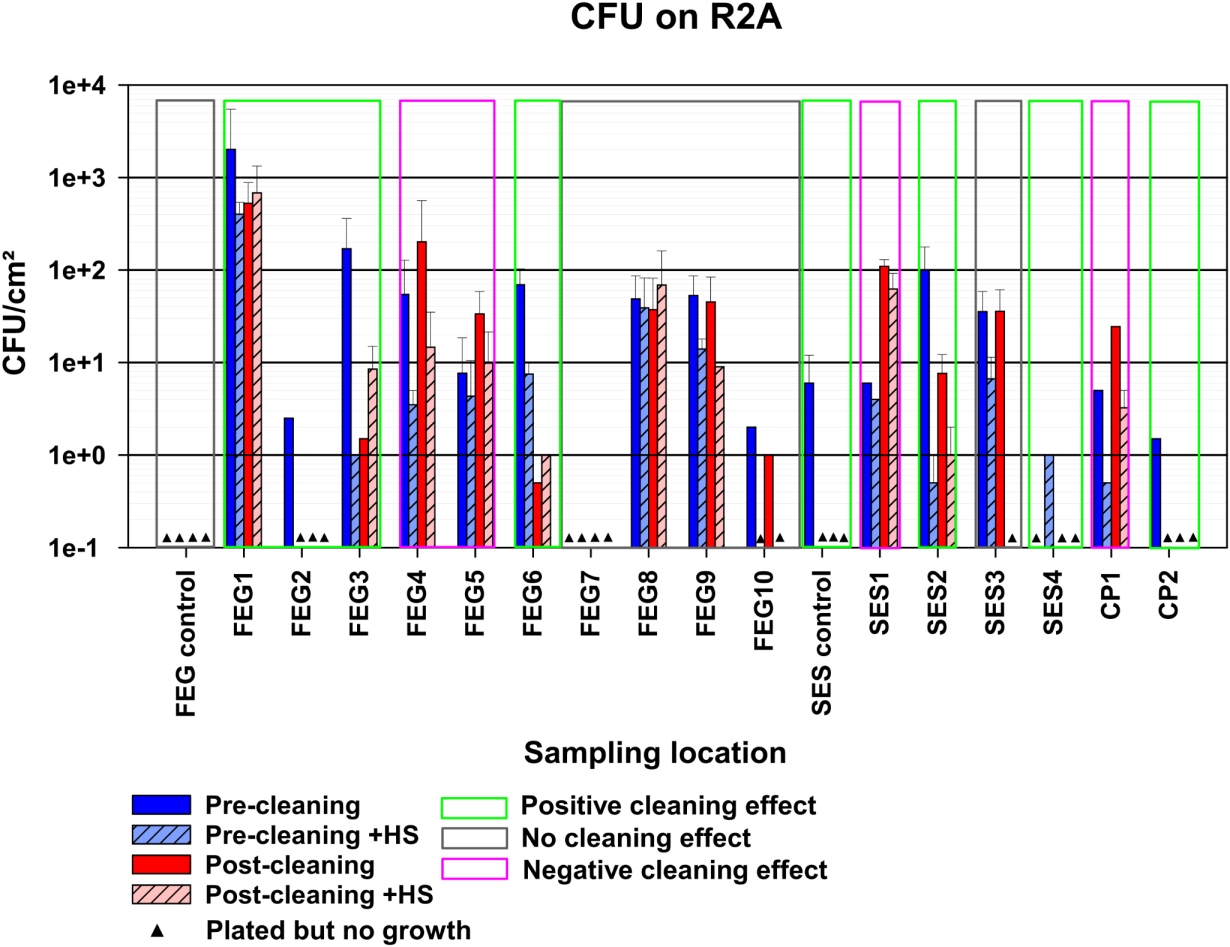
Observed bacterial bioburden of the pre- and post-cleaning samples. The average CFU/cm² of the samples plated on R2A, calculated by taking the average of the CFU/cm² of each plate with visible growth for each condition. The error bars represent the standard deviation of the calculated values. Samples without any visible growth are represented by black triangles. Depending on the reduction in bioburden observed after cleaning, compared to before, samples were classified as possessing either a positive, negative, or no cleaning effect, indicated by the green, red, or gray box, respectively. Due to the number of available samples, each bar represents a single replicate.

The FEG control samples on R2A showed no growth under all tested conditions (**Figure 3**). The SES control samples, on the other hand, showed a bioburden of 6 ± 6 CFU (colony forming units)/cm² for the pre-cleaning without heat shock condition. No CP control samples were available. The bioburden of the different sampling positions before cleaning without heat shock treatment varied greatly. The highest bioburden was found at FEG1 with 517 ± 226 CFU/cm², while the FEG7 and SES4 samples showed no growth. Exposing the samples to heat shock treatment before cultivation, to eliminate most vegetative cells while keeping spores intact, led to a decreased bioburden in most cases. The only exceptions were SES1, where the differences in bioburden were minor, and SES4, where growth was only observed after heat shock. In all sampling positions besides FEG5, FEG7, SES1, SES3, and CP1, the post-cleaning samples showed a clear decrease in bioburden after cleaning. The remaining positions either showed no differences in the case of FEG7 and SES3 or an increase in bioburden in the case of FEG5, SES1, and CP1. Heat shock treatment of the post-cleaning samples showed increased bioburdens for FEG3.

### 3.2 Heat shock resistance

**Figure 4:**
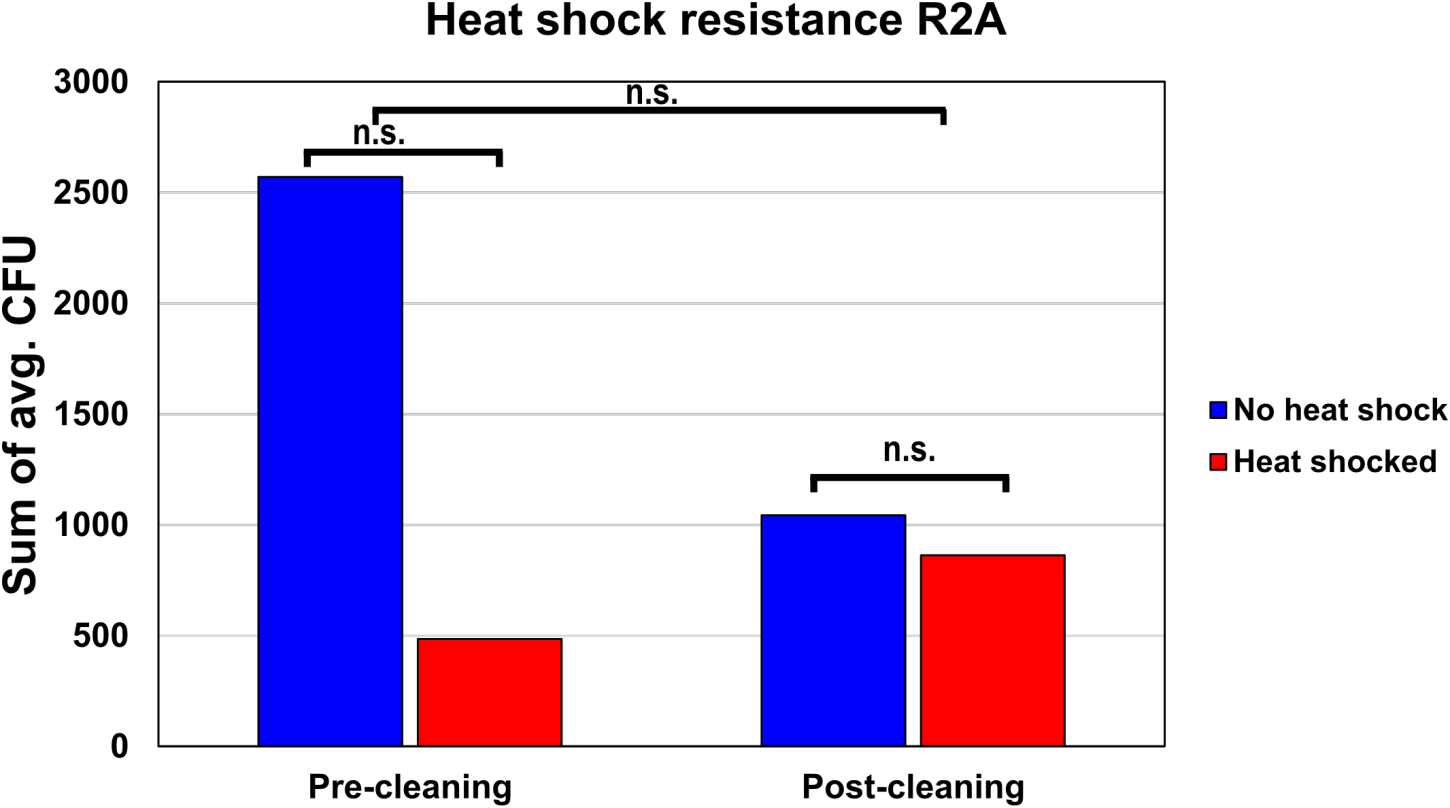
Heat shock resistance of the pre- and post-cleaning samples. To estimate the fraction of organisms with heat shock resistance, the average CFUs of all pre-cleaning and post-cleaning samples either before or after heat shock were summed up and compared to each other.

The average amount of heat shock-resistant organisms found before and after cleaning was evaluated on R2A. In the pre-cleaning samples, the average CFU declined from 2,571 before heat shock to 484 after heat shock, resulting in a fraction of heat shock-resistant organisms of 18.9% (**Figure 4**). The post-cleaning samples showed 1,043 CFU before heat shock and 863 CFU after heat shock, resulting in a fraction of heat shock-resistant organisms of 82.7%. A two-way ANOVA showed that these differences were not significant, as the *p*-values for the cleaning, heat shock, and combined effect were 0.583, 0.566, and 0.728, respectively.

### 3.3 Most abundant organisms identified by NGS

The analysis of the NGS data revealed a vast variety of bacterial genera in both the pre- and post-cleaning samples, as well as the FEG9 samples from the second campaign. The following section will focus on the 20 most abundant genera.

The most dominant genus in both pre- and post-cleaning samples (**Table 1**) was *Candidatus Profftella*. It accounted for 18.1% of all identified amplicon sequence variants (ASVs) in the pre-cleaning samples and 17.6% after cleaning. *Pseudomonas* species were also highly abundant, accounting for the second highest fraction of reads in both pre- and post-cleaning samples with 7.74% and 16.1%, respectively. Bacteria belonging to the *Ralstonia* genus accounted for 5.80% of ASVs in the pre-cleaning samples, making it the third most abundant in the pre-cleaning samples, while only accounting for 1.73% of ASVs in the post-cleaning samples. Other organisms that accounted for large percentages of the total ASVs in either the pre- or post-cleaning samples belonged to *Acinetobacter*, *Zooglea*, and *Glutamicibacter*. The remaining groups of organisms each accounted for less than 4% of all ASVs of a specific sampling condition. Among those, *Rhodococcus* stood out by accounting for the third highest number of total reads, while accounting for only 3.63% of ASVs in the pre-cleaning samples and 3.36% of ASVs in the post-cleaning samples. The most common groups of organisms in the pre- and post-cleaning samples differed in their presence in the percentage of different sampling positions. In the pre-cleaning samples, *Candidatus Profftella* was present in 95% of sampling positions, making it the most widely distributed genus.

**Table 1:** Most abundant genera—pre- and post-cleaning.

Genus	Fraction of reads—pre-cleaning (%)	Fraction of reads—post-cleaning (%)	Present in % of samples—pre-cleaning (%)	Present in % of samples—post-cleaning (%)
*Candidatus Profftella*	18.1	17.6	95	70
*Pseudomonas*	7.74	16.1	90	90
*Rhodococcus*	3.63	3.36	65	60
*Acinetobacter*	2.11	4.56	65	90
*Ralstonia*	5.80	1.73	60	40
*Enhydrobacter*	3.85	1.06	35	45
*Zooglea*	0.54	4.17	25	65
*Glutamicibacter*	4.00	0.88	65	35
*Brevundimonas*	2.56	2.07	60	70
*Cutibacterium*	2.72	1.61	60	70
*Flavobacterium*	0.77	2.83	40	55
*Staphylococcus*	2.67	1.10	55	50
*Nakamurella*	1.61	2.00	50	55
*Herbaspirillum*	3.29	0.94	50	50
*Chryseobacterium*	0.84	1.91	30	35
*Phyllobacterium*	3.25	0.08	35	5
*Mycobacterium*	1.50	0.95	45	45
*Microbacterium*	1.85	0.48	70	25
*Corynebacterium*	1.46	0.81	55	40
*Acidovorax*	0.27	1.61	15	40

The 20 genera with the highest number of total reads from the pre- and post-cleaning samples were compared by the percentage of reads from their respective sampling condition they accounted for, as well as the percentage of sampling positions where they accounted for 10 or more reads under each corresponding sampling condition.

However, in the post-cleaning samples, it was found in only 70% of all samples, which made it the third most widely distributed genus in the post-cleaning samples after *Pseudomonas* and *Acinetobacter*. *Pseudomonas* was present in 90% of all sampling positions in both cases, while *Acinetobacter*, which was also present in 90% of post-cleaning samples, was only found in 65% of pre-cleaning samples. All other genera were not found in more than 70% of samples for both conditions. *Phyllobacterium* was also notable in its distribution, as it was found in only a single post-cleaning sample.

For the second campaign, the most abundant group of organisms in the FEG9 samples (**Table 2**) was bacteria of the *Citricoccus* genus, accounting for 13.8% of all ASVs, and *Pseudomonas*, which accounted for 13.3% of all ASVs. They were followed by *Staphylococcus*, *Brevundimonas*, *Rhodococcus*, and *Acinetobacter*, which accounted for 9.22%, 6.91%, 4.95%, and 4.33%, respectively. The remaining genera all accounted for less than 4% of the total reads. *Brevundimonas* was the most widely distributed genus, being found in 83% of all samples. *Pseudomonas* was a close second with a presence in 79%, followed by *Citricoccus*, which was found in 67%, and *Staphylococcus*, which was found in 63% of all samples. All other genera were present in 50% or less of all samples.

**Table 2:** Most abundant genera—FEG9.

Genus	Fraction of total reads—FEG9 (%)	Present in % of samples—FEG9 (%)
*Citricoccus*	13.8	67
*Pseudomonas*	13.3	79
*Staphylococcus*	9.22	63
*Brevundimonas*	6.91	83
*Rhodococcus*	4.95	50
*Acinetobacter*	4.33	38
*Flavobacterium*	3.94	42
*Nesterenkonia*	3.26	25
*Cutibacterium*	1.92	38
*Enhydrobacter*	1.53	13
*Candidatus Profftella*	1.48	38
*Microbacterium*	1.44	33
*Chryseobacterium*	1.35	46
*Sphingobium*	1.21	25
*Paracoccus*	1.17	33
*Nocardia*	1.12	13
*Stenotrophomonas*	1.11	33
*Corynebacterium*	1.09	25
*Brevibacterium*	1.03	25
*Massilia*	1.01	21

The 20 genera with the highest combined number of reads from the FEG9 samples were compared by the percentage of total reads they accounted for and the percentage of sampling positions where they accounted for 10 or more reads in each corresponding sample.

### 3.4 Distribution of genera in each sample

**Figure 5:**
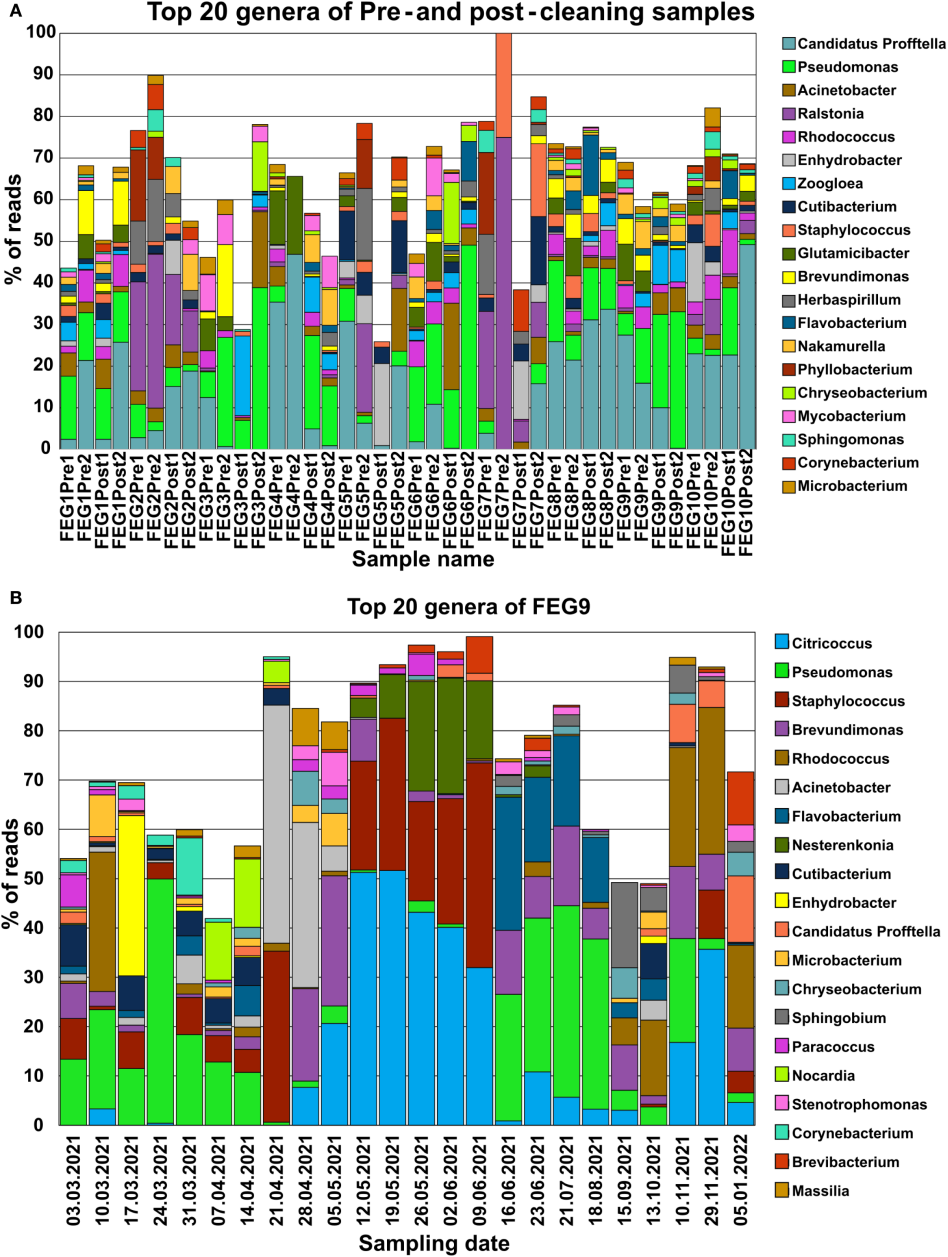
Microbiome composition of each individual sample determined through NGS. The microbiome composition of each sample from the pre- and post-cleaning samples **(A)** and the FEG9 samples **(B)** was visualized by displaying the fraction of reads that each of the 20 most abundant genera made up for the respective sample in percent. All remaining genera were left out to prevent overcrowding of the graph.

*Candidatus Profftella* was present in almost all pre- and post-cleaning samples, with the only exceptions being both post-cleaning samples of FEG3, FEG6 Post2, and the FEG7 Pre2 and Post1 samples (**Figure 5A**). *Pseudomonas* species were similarly widely distributed, being present in all samples besides FEG4 Pre1, FEG5 Post1, and again FEG7 Pre2 and Post1. Both organisms made up a large percentage of reads in multiple samples. Another widely distributed genus was *Acinetobacter*, which was present at relatively low levels in most samples, but reached a relative abundance higher than 10% in FEG3 Post2, FEG5 Post2, and FEG6 Post1. Other genera were not as widely distributed but had relatively high abundance levels in some samples. Examples included *Ralstonia*, which accounted for large portions of the total reads in all FEG2 samples, FEG5 Pre2, and both FEG7 pre-cleaning samples, as well as relatively high abundance levels of *Herbaspirillum* and *Phyllobacterium* in most cases. The *Zooglea* genus was the most abundant in FEG3 Pre2, but had an abundance of 10% or below in all other samples.

In the second campaign, the first seven FEG9 samples showed a significant relative abundance of *Pseudomonas*. Additionally, various genera like *Rhodococcus* or *Enhydrobacter* were also highly abundant in single samples (**Figure 5B**). *Staphylococcus* was also present at various levels in most of these samples. On 21 April 2021, *Pseudomonas* almost completely disappeared. Instead, *Staphylococcus* and *Acinetobacter* accounted for most of the reads.

However, on 28 April 2021, *Staphylococcus* was suddenly not present anymore, while *Brevundimonas*, *Citricoccus*, and *Brevundimonas* were more abundant. The relative abundance of these three genera increased on 05 May 2021, while the relative abundance of *Acinetobacter* declined drastically. The sample from 12 May 2021 differed greatly from the previous week, with *Citricoccus* being the most dominant genus, with a relative abundance of more than 50%, and *Staphylococcus* being present again with a relative abundance of 22%. From 12 May 2021 to 09 June 2021, the abundance of *Citricoccus* continuously declined, while the abundance of *Staphylococcus* and *Nesterenkonia* fluctuated but accounted for most of the remaining reads. The next large shift in microbiome composition occurred from 09 June 2021 to 16 June 2021, where *Citricoccus*, *Staphylococcus*, and *Nesterenkonia* were found only in small amounts or not at all. The following samples were instead mostly characterized by *Pseudomonas*, *Flavobacterium*, and *Brevundimonas*. The microbiome composition of FEG9 continued to shift between samples; however, *Rhodococcus* played a dominant role in the last 3 months.

### 3.5 Alpha diversity analysis

To analyze the alpha diversity of the samples, the Chao1, inverse Simpson, and Shannon indices were calculated.

**Figure 6:**
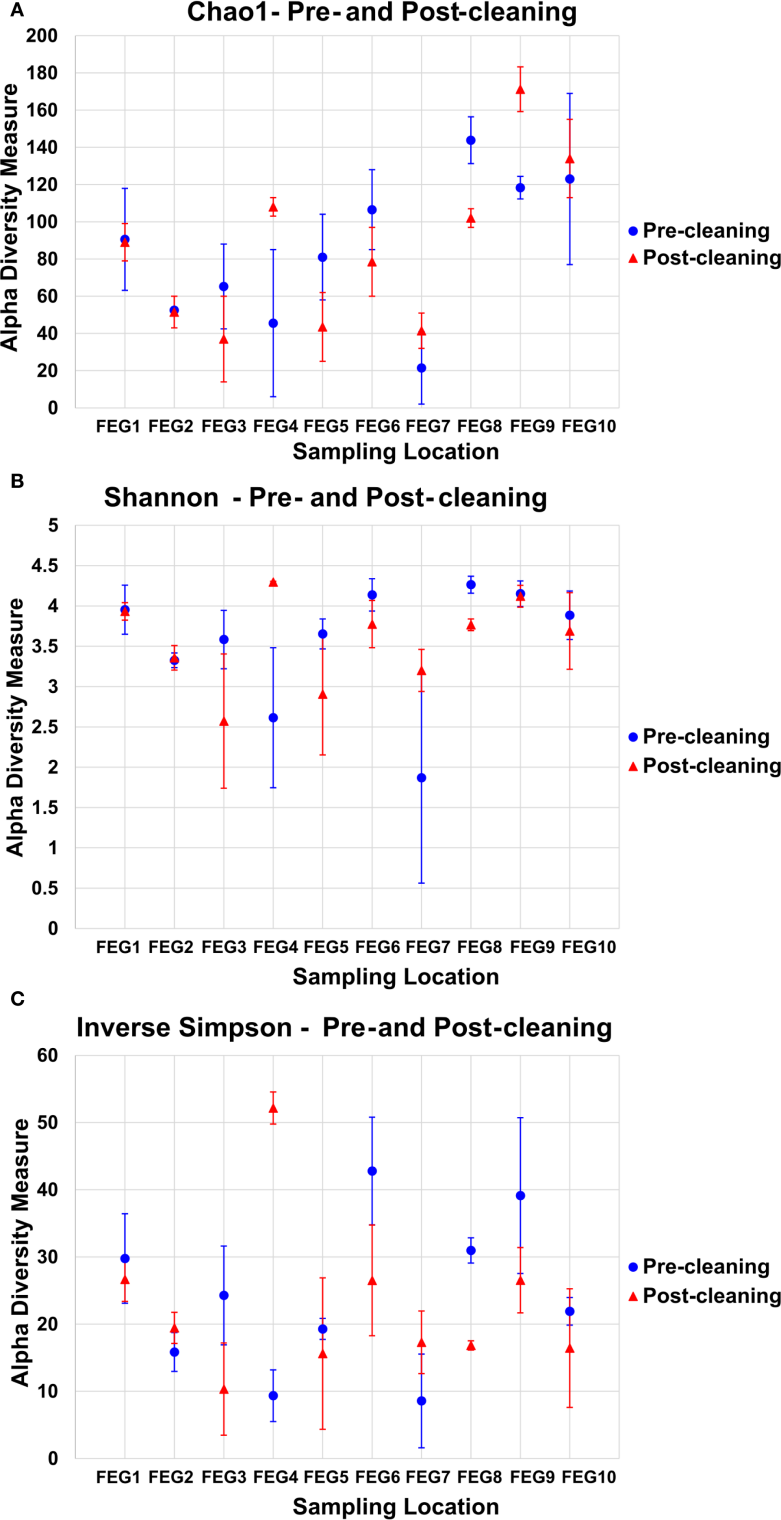
Alpha diversity measures of the pre- and post-cleaning samples. To analyze the alpha diversity of the pre- and post-cleaning samples, the Chao1 index **(A)**, the Shannon index **(B)**, and the inverse Simpson index **(C)** were calculated, and the average value of both samples was plotted for each sampling location. The error bars represent the standard deviation of the diversity measure values of both samples.

**Figure 7:**
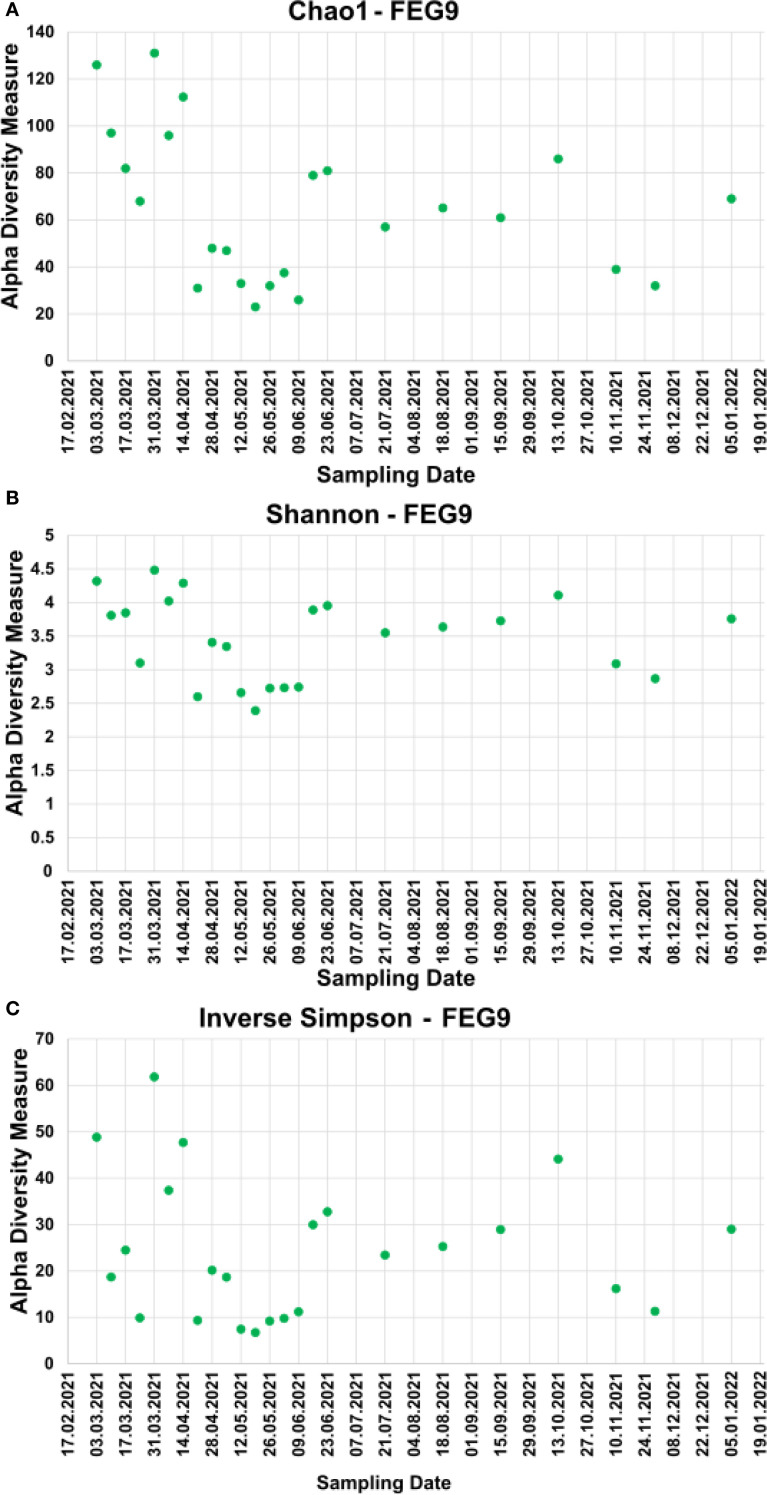
Alpha diversity measures of the FEG9 samples. To analyze the alpha diversity of the FEG9 samples, the Chao1 index **(A)**, the Shannon index **(B)**, and the inverse Simpson index **(C)** were calculated and plotted against the sampling date.

For the pre- and post-cleaning samples, the Chao1 index (**Figure 6A**) ranged from 19.5 ± 9.5 for the pre-cleaning FEG7 samples to 171.2 ± 12.0 for the FEG9 post-cleaning samples. Only very minor changes were observed between pre- and post-cleaning for the FEG1, FEG2, and FEG10 samples. An increase in average Chao1 after cleaning could be seen for the FEG4, FEG7, and FEG9 samples. The FEG3, FEG5, FEG6, and FEG8 samples, on the other hand, exhibited decreased values after cleaning. The Chao1 index of the FEG9 samples (**Figure 7A**) ranged from a minimum of 23, observed on 19 May 2021, to a maximum of 131, observed on 31 March 2021. The Chao1 index started out high with a value of 126 on 03 March 2021, but declined over the next 3 weeks to a value of 68. It subsequently increased to 131 on 31 March 2021. The following 2 weeks showed values of 96 and 112, respectively. Afterward, the observed community richness plummeted. For the period from 21 April 2021 to 09 June 2021, the Chao1 index ranged from 26 to 48. On 23 June 2021, it jumped to a value of 79, and the community richness in the period until 13 October 2021 fluctuated between 57 and 86. Toward the end of the observed period, the Chao1 index once again plummeted to a value of 39 on 10 November 2021, followed by a value of 32 on 29 November 2021, but it rose again to 69 on the last sampling date on 05 January 2022.

The values of the Shannon index of the pre- and post-cleaning samples (**Figure 6B**) ranged from 1.87 ± 1.31 for the pre-cleaning FEG7 samples to 4.29 ± 0.01 for the post-cleaning FEG4 samples. For the FEG1, FEG2, FEG9, and FEG10 samples, no drastic changes in microbial diversity between pre- and post-cleaning could be observed. This was not the case for the FEG3, FEG5, FEG6, and FEG8 samples that saw a noticeable decrease in diversity after cleaning, while FEG4 and FEG7 showed a significantly increased Shannon index in the post-cleaning samples. The Shannon index values of the FEG9 samples (**Figure 7B**) were all in the range of 2.39, observed on 19 May 2021, to 4.48, measured on 31 March 2021. The first sample showed a Shannon index of 4.32, which decreased over the next 3 weeks to 3.10, which was followed by a jump up to 4.48 on 31 March 2021. The Shannon index remained higher than 4 for the next 2 weeks, but then fell rapidly to 2.60 on 21 April 2021. The following 2 weeks showed increased values of 3.41 and 3.34, which was once again followed by a period of low microbial diversity, with values fluctuating between 2.39 and 2.74 from 12 May 2021 to 09 June 2021. The subsequent period from 16 June 2021 to 13 October 2021 showed noticeably higher Shannon indices between 3.55 and 4.11. Between 13 October 2021 and 10 November 2021, the microbial diversity once again plummeted, with Shannon indices of 3.09 and 2.87 on 10 November 2021 and 29 November 2021, respectively, before rising again toward the last sampling date, with a value of 3.76 on 05 January 2022.

The inverse Simpson index (**Figure 6C**) of the pre- and post-cleaning samples ranged from 8.58 ± 6.98 for the pre-cleaning FEG7 samples to 52.16 ± 2.39 for the FEG4 post-cleaning samples. The FEG1, FEG2, FEG5, and FEG10 samples exhibited small differences between the pre- and post-cleaning samples. Much more pronounced differences could be observed for the FEG2, FEG4, and FEG7 samples, where the inverse Simpson index increased after cleaning, as well as for the FEG3, FEG6, FEG8, and FEG9 samples, which showed lower values after cleaning. From there, it rose to 24.5 and then fell again to 9.92 in the next 2 weeks. Afterward, there was a sudden jump to 61.6 on 31 March 2021, followed by lower values of 37.4 and 47.4. The inverse Simpson index then plummeted to 9.37 on 21 April 2021, but increased again to approximately 20 for the next 2 weeks. It then dropped again and fluctuated between 6.75 and 11.2 for the next 5 weeks. Between 09 June 2021 and 16 June 2021, there was another jump up to 30.0, from which the inverse Simpson index ranged from 23.4 and 32.8 for the period of 16 June 2021 to 15 September 2021. On 13 October 2021, there was a rise to 44.1, which was followed by a drop to 16.2 and further to 11.3 on the following two sampling dates and finally a rise to 29.0 on 05 January 2022.

The inverse Simpson index of the FEG9 samples (**Figure 7C**) ranged from 6.75 on 19 May 2021 to 61.8 on 31 March 2021. The first observed value was 48.8 on 03 March 2021, which was immediately followed by a sharp drop to 18.7 in the next week. From there, it rose to 24.5 and then fell again to 9.92 in the next 2 weeks. Afterward, there was a sudden jump to 61.8 on 31 March 2021, followed by lower values of 37.4 and 47.4. The inverse Simpson index then plummeted to 9.37 on 21 April 2021, but increased again to approximately 20 for the next 2 weeks. It then dropped again and fluctuated between 6.75 and 11.2 for the next 5 weeks. Between 09 June 2021 and 16 June 2021, there was another jump up to 30.0, from which the inverse Simpson index ranged from 23.4 and 32.8 for the period of 16 June 2021 to 15 September 2021. On 13 October 2021, a rise to 44.1 was seen, which was followed by a drop to 16.2 and further to 11.3 on the following two sampling dates and finally a rise to 29.0 on 05 January 2022.

### 3.6 Beta diversity analysis

The beta diversity of the samples was analyzed by calculating the Bray–Curtis dissimilarity and visualized with non-metric multidimensional scaling (NMDS) and principal component analysis (PCoA).

**Figure 8:**
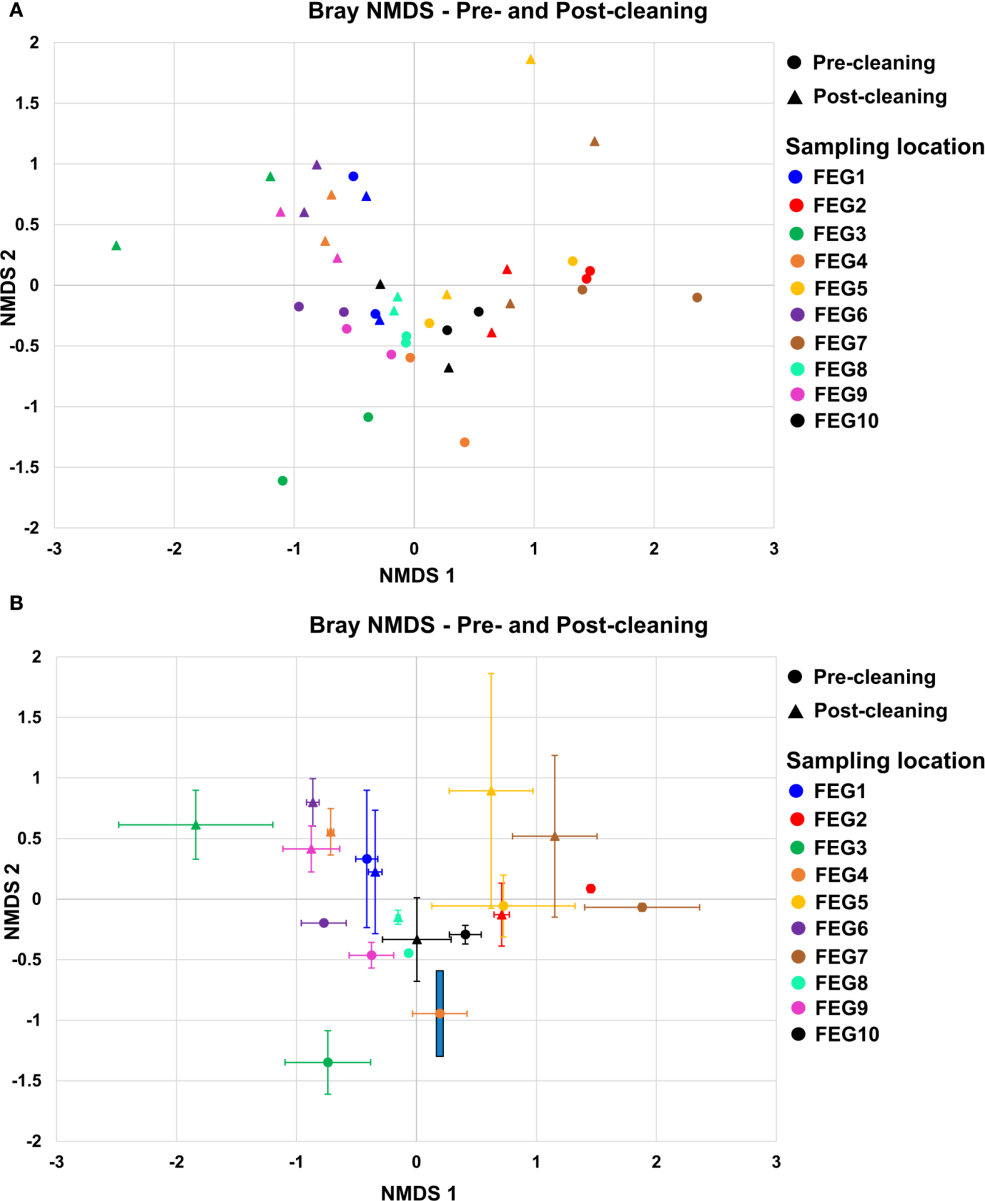
Beta diversity. The beta diversity of the pre- and post-cleaning samples **(A)** and the FEG9 samples **(B)** was calculated using the Bray–Curtis NMDS and plotted with NMDS1 on the *x*-axis and NMDS2 on the *y*-axis. The error bars represent the averages of both duplicates of each sampling position.

For the pre-cleaning samples (**Figure 8A**), the NMDS 1 component ranged from −0.77 ± 0.19 for the FEG6 samples to 1.88 ± 0.48 for the FEG7 samples. For the post-cleaning samples, it ranged from −1.84 ± 0.64 for the FEG3 samples to 1.15 ± 0.35 for the FEG7 samples. The NMDS2 component for the pre-cleaning samples, on the other hand, ranged from −1.35 ± 0.26 for the FEG3 samples to 0.33 ± 0.57 for the FEG1 samples. For the post-cleaning samples, these values ranged from −0.33 ± 0.34 for the FEG10 samples to 0.89 ± 0.97 for the FEG5 samples. Overall, most samples were in the range of −1.0 to 1.0 for both NMDS1 and NMDS2. The number of samples was too low to calculate *p*-values for the differences between each sampling condition with pairwise permutational multivariate analysis of variance (PERMANOVA). However, pairwise PERMANOVA for the sampling locations showed that most differences between locations were not significant (*p* > 0.05) (**Supplementary Table S1**). FEG7 was significantly different from most other sampling locations, as it only showed *p*-values above 0.05 when compared to FEG2, FEG3, and FEG5. The difference between pre- and post-cleaning samples was not significant with a *p*-value of 0.142. The PCoA of the pre- and post-cleaning samples (**Supplementary Figure S1**) showed that PCoA1 accounted for 15.5% of the observed differences, while PCoA2 accounted for 13.8%. The samples formed two tighter groups, but a lot of samples were also loosely distributed across the plot. The first one was found in the upper right quadrant with values for both PCoA1 and PCoA2 between 0.2 and 0.4 and contained mostly post-cleaning samples, with the exception of FEG1 Pre1. A second cluster could be seen in the lower half of the graph, with PCoA1 values between −0.1 and 0.2 and PCoA2 values between −0.05 and −0.35. The upper left quadrant contained multiple samples with PCoA2 values approximately 0.2, but their PCoA1 ranged from −0.1 to −0.5. Only a single sample, FEG3 Pre1, was observed near the origin of the plot.

For the first 7 weeks, the FEG9 samples (**Figure 8B**) ranged mostly between 1.0 and 1.5, with the biggest exception on 10 March 2021, with a value of 0.68. The NMDS2 component varied more strongly, ranging from −0.35 on 03 March 2021 to 1.81 on 24 March 2021. On 21 April 2021, there was a large shift in NMDS1, going to a value of −0.62 compared to 1.10 in the previous week. For the following 7 weeks, the NMDS1 component was relatively similar, ranging from −1.34 to −0.81, while the NMDS2 component ranged from −0.49 to 0.74. On 16 June 2021, there was another shift. The NMDS1 jumped to 0.33, with an NMDS2 of −0.79, and for the following three sampling dates, both NMDS1 and NMDS2 were in a similar range, with NMDS1 ranging from −0.07 to 0.52 and NMDS2 ranging from −0.94 to −0.23. The following sample from 15 September 2021 was in a similar range for NMDS1 with a value of −0.07, but its NMDS2 was noticeably lower with a value of −1.38. The next sample from 13 October 2021 was also significantly different with an NMDS1 of 1.13 and an NMDS2 of −0.84. Following this, there was another drastic change in the NMDS1 on 11 October 2021, going to −0.37. The sample from 29 November 2021 was relatively similar to it with an NMDS1 of −0.93 and an NMDS2 of −0.17, while the last sample from 05 January 2022 saw another large shift in NMDS2, going to −1.31. As there was only one sample from each sampling date, pairwise PERMANOVA was not applicable. Instead, the Spearman correlation between the sampling date and both NMDS components was calculated. Spearman’s rho for the first component was −0.377 with a *p*-value of 0.070, while it was −0.623 with a *p*-value of 0.014 for the second component.

The PCoA of the FEG9 samples (**Supplementary Figure S2**) showed that PCoA1 accounted for 22.7% and PCoA2 for 12.7% of the observed diversity. The samples formed three distinct clusters. Most of the samples from the beginning of the sampling period were found in the upper left quadrant with PCoA1 values between −0.1 and −0.35 and PCoA2 values between 0.1 and 0.3. Samples from the middle of the sampling period clustered in the upper right quadrant with PCoA1 values of approximately 0.5 and PCoA2 values between 0 and 0.1. Most of the samples from the tail end of the sampling period were found in the lower half of the plot with PCoA1 values ranging from −0.3 to 0.25 and PCoA2 values between −0.1 and −0.4.

### 3.7 Sequencing of pre- and post-cleaning isolates

The most common identified isolates in the pre- and post-cleaning samples belonged to *Paenibacillus*, *Bacillus*, and *Fictibacillus*, which together accounted for more than one-third of the 193 total isolates (**Table 3**). They were followed by *Rossellomorea* and *Peribacillus*, which were identified 17 and 16 times, respectively. Twelve isolates were classified as *Staphylococcus*, while *Micrococcus* and *Buttiauxella* were identified 11 times. All the other identified genera were identified in less than 10 isolates.

**Table 3:** Genera identified in isolates—pre- and post-cleaning.

Genus	Number of isolates
*Paenibacillus*	28
*Bacillus*	26
*Fictibacillus*	23
*Rossellomorea*	17
*Peribacillus*	16
*Staphylococcus*	12
*Micrococcus*	11
*Buttiauxella*	11
*Gordonia*	8
*Microbacterium*	7
*Rhodococcus*	6
*Kocuria*	4
*Alkalihalobacillus*	3
*Gottfriedia*	3
*Cytobacillus*	3
*Ralstonia*	2
*Brachybacterium*	2
*Paracoccus*	2
*Nesterenkonia*	2
*Enhydrobacter*	1
*Moraxella*	1
*Niallia*	1
*Priestia*	1
*Aerococcus*	1
*Phyciococcus*	1
*Williamsia*	1

Cultivation of the pre- and post-cleaning samples yielded 193 different isolates that were identified via partial 16S rRNA gene sequencing.

### 3.8 Sequencing of FEG9 isolates

The sequencing of the partial 16S rRNA gene of the 69 FEG9 isolates revealed that the highest number of isolates belonged to the genera *Paenibacillus* and *Bacillus*, with 11 and 10 isolates, respectively (**Table 4**). Other genera that were identified five times or more were *Chryseobacterium*, which was identified six times, and *Rhodococcus*, *Microbacterium*, and *Pseudomonas*, which were identified five times.

**Table 4:** Genera identified in isolates—FEG9.

Genus	Number of isolates
*Paenibacillus*	11
*Bacillus*	10
*Chryseobacterium*	6
*Pseudomonas*	5
*Rhodococcus*	5
*Microbacterium*	5
*Flavobacterium*	4
*Fictibacillus*	3
*Nakamurella*	3
*Agrobacterium*	2
*Psychrobacillus*	2
*Sanguibacter*	2
*Citricoccus*	1
*Buttiauxella*	1
*Williamsia*	1
*Herbiconiux*	1
*Rossellomorea*	1
*Exiguobacterium*	1
*Acinetobacter*	1
*Hymenobacter*	1
*Methylorubrum*	1
*Cohnella*	1
*Variovorax*	1

Cultivation of the FEG9 samples yielded 69 different isolates that were identified via partial 16S rRNA gene sequencing.

### 3.9 Fraction of potential spore formers

**Figure 9:**
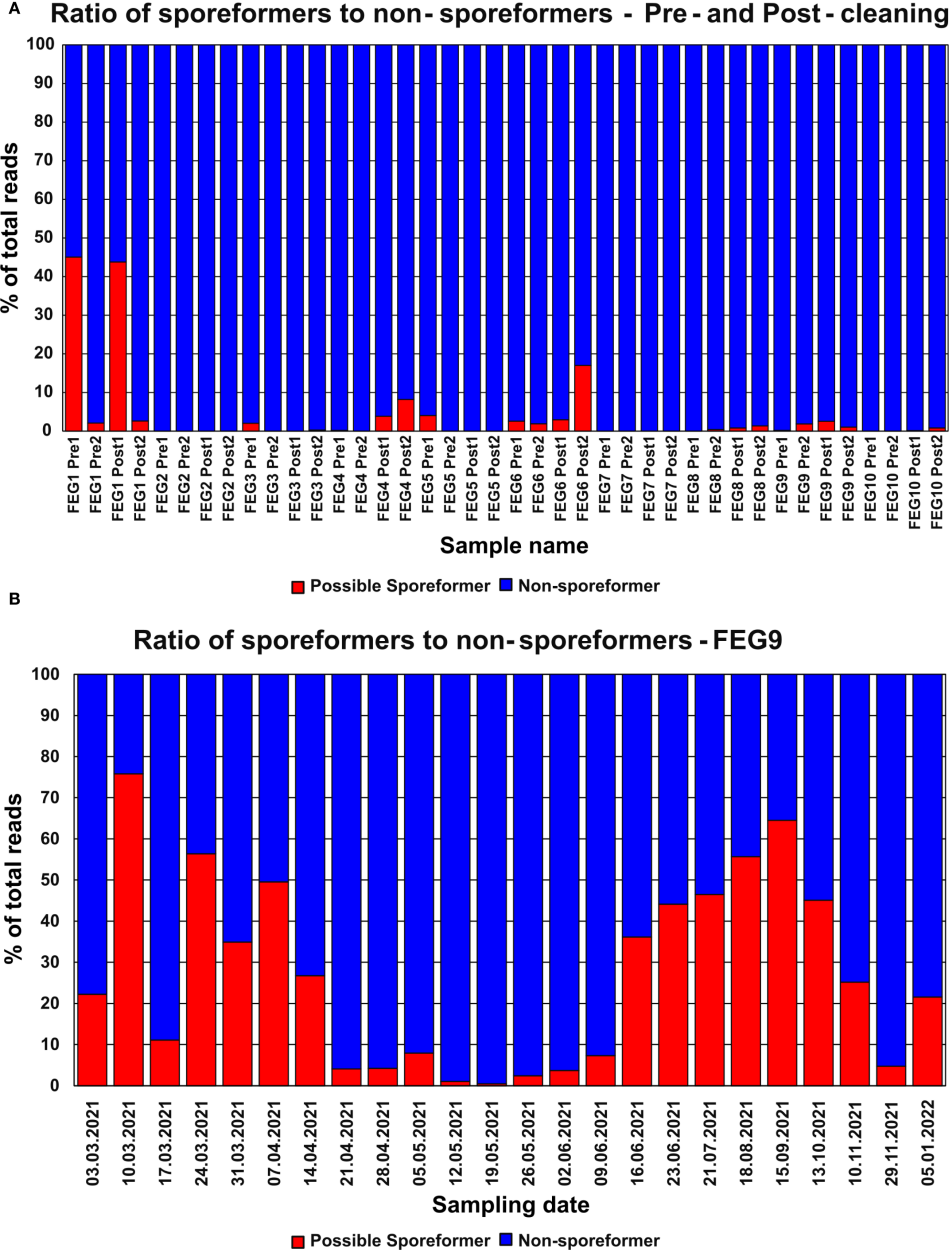
Abundance of organisms with the potential to form spores—NGS. The relative abundance of genera with the potential to form spores identified through NGS in the pre- and post-cleaning samples **(A)** and the FEG9 samples **(B)** was calculated for each individual sample.

Most pre- and post-cleaning samples contained next to no genera with the potential to form spores (**Figure 9A**). Notable exceptions were the FEG1 Pre1 and FEG1 Post1 samples with 45.1% and 43.8% of potential spore formers, respectively, as well as FEG4 Post2 with 8.18% and 17.0%. All the other samples showed fractions of potential spore formers well below 5%, and in many cases, none were detected at all, with the average fraction of potential spore formers of all samples being 3.64%.

The highest fraction of organisms with the potential to form spores in a sample from the second campaign was observed on 10 March 2021 with 75.3%, which was a significant increase from the fraction of 22.2% observed in the previous week (**Figure 9B**). This was followed by a large drop to 11.1% on 17 March 2021. From 24 March 2021 to 14 April 2021, the fraction of potential spore formers fluctuated between 26.8% and 56.3%. Following this, the fraction of potential spore formers remained below 10% for the period from 21 April 2021 to 09 June 2021. Afterward, the fraction increased continuously and peaked on 15 September 2021 with a value of 64.5%, but then declined over the next three sampling dates to 4.74% on 29 November 2021. The last sampling date on 05 January 2022 saw a moderate increase to 21.6%. The average fraction of potential spore formers was 27.1%.

Analyzing the fraction of potentially spore-forming organisms in the isolates pre- and post-cleaning for each sampling position revealed that all sampling sites from the CP and the SES, except for CP2, saw significant increases after cleaning (**Table 5**). Similar increases in the post-cleaning samples could be observed for FEG1 and FEG10. For FEG3 and FEG4, the relative frequencies of potentially spore-forming organisms increased to 66.7% and 76.9%, respectively, whereas the relative frequencies for FEG5, FEG6, FEG8, and FEG9 all showed a strong decrease in potentially spore-forming organisms after cleaning. The largest decreases were observed for FEG5, going from 100% to 25%, and FEG9, going from 77.8% to 14.3%. FEG2 showed no potential spore-forming organisms both before and after cleaning, while FEG7 did not show any viable isolates at all. The average fraction of potential spore-forming organisms of all samples combined remained largely unchanged between pre- and post-cleaning, going from 72.0% to 74.1%. The relative frequency of genera with the potential to form spores in the FEG9 isolates was 65.2%, which was slightly lower than what was observed for the pre- and post-cleaning isolates.

**Table 5:** Fraction of spore-forming organisms in the isolates.

Sampling site	Sampling campaign	Fraction of potential spore-forming organisms (%)
CP1	Pre-cleaning	50
	Post-cleaning	100
CP2	Pre-cleaning	0
	Post-cleaning	0
SES1	Pre-cleaning	50
	Post-cleaning	95.5
SES2	Pre-cleaning	94.1
	Post-cleaning	100
SES3	Pre-cleaning	83.3
	Post-cleaning	100
SES4	Pre-cleaning	0
	Post-cleaning	100
FEG1	Pre-cleaning	87.5
	Post-cleaning	100
FEG2	Pre-cleaning	0
	Post-cleaning	0
FEG3	Pre-cleaning	46.2
	Post-cleaning	66.7
FEG4	Pre-cleaning	50
	Post-cleaning	76.9
FEG5	Pre-cleaning	100
	Post-cleaning	25
FEG6	Pre-cleaning	90
	Post-cleaning	50
FEG7	Pre-cleaning	0
	Post-cleaning	0
FEG8	Pre-cleaning	84.6
	Post-cleaning	71.4
FEG9	Pre-cleaning	77.8
	Post-cleaning	14.3
	FEG9 sampling	65.2
FEG10	Pre-cleaning	50
	Post-cleaning	100
Average of all samples	Pre-cleaning	72
	Post-cleaning	74.1
	FEG9 sampling	65.2

The fraction of potential spore-forming organisms in the isolates was calculated for each sampling site from each sampling campaign. The average fraction of all samples from each campaign was calculated as well.

### 3.10 Comparison of genera identified from the cultivated isolates and from the surface samples

**Figure 10:**
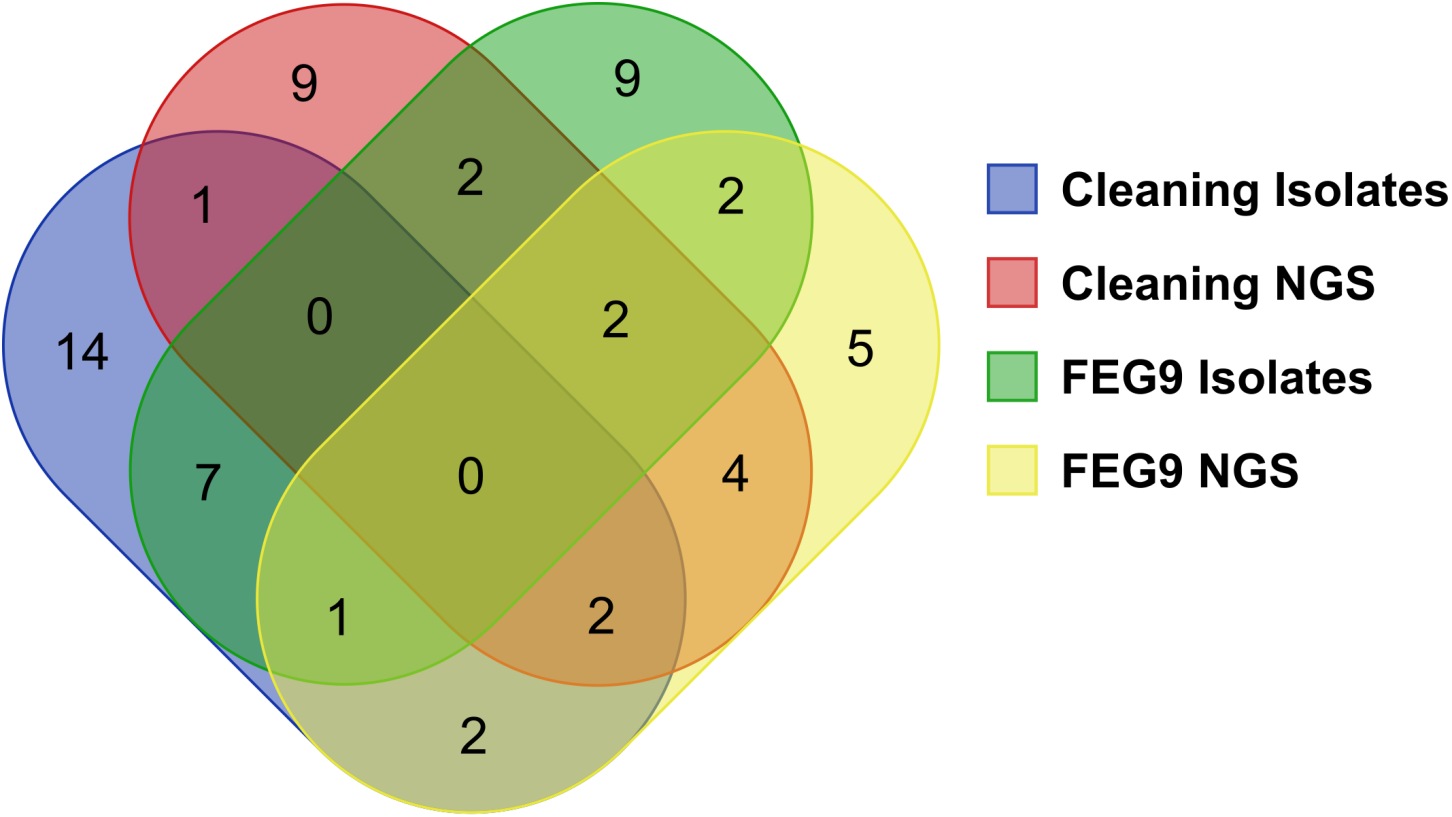
Comparison of genera identified through NGS and direct sequencing of isolates. To compare the overlap of genera between the different sequencing approaches, a Venn diagram was constructed with the following categories: genera found in the pre- and post-cleaning samples through the sequencing of isolates (cleaning isolates), genera found in the pre- and post-cleaning samples through NGS of surface samples (cleaning NGS), genera found in the FEG9 samples through the sequencing of isolates (FEG9 isolates), and genera found in the FEG9 samples through NGS of surface samples (FEG9 NGS).

The different groups of samples were compared to each other in regard to the number of identified genera shared between groups (**Figure 10**). Twenty-seven different genera were identified through the sequencing of the isolates of the pre- and post-cleaning samples (cleaning isolates), while the 20 most abundant genera found in the surface samples from the pre- and post-cleaning samples (cleaning NGS) were used for the comparison. The number of genera shared between these two groups was much lower, with only five genera being found in both groups. The sequencing of the FEG9 isolates revealed 23 different genera, while the 20 most common genera from the FEG9 surface samples (FEG9 NGS) were used.

These two groups shared seven genera between them. The FEG9 isolates and the cleaning isolates shared eight genera between them, with two genera also being identified in FEG9 NGS.

FEG9 NGS and cleaning NGS shared 12 genera, with four of them also being found in cleaning isolates and another six in FEG9 isolates. Cleaning isolates and FEG9 NGS shared two genera that were not found in the other groups, while cleaning NGS and FEG9 isolates shared a single genus that was not found in the previous two groups. *Rhodococcus* and *Microbacterium* were the two genera that were shared between all four categories.

### 3.11 Prediction of gene functions from 16S rRNA sequences

To further investigate the potential presence of pathogenic microorganisms in the EDEN ISS microbiome, a prediction of gene functions from the 16S rRNA sequences was carried out with Picrust2, followed by annotation with the KEGG BRITE database.

In total, 8,136 KEGG orthologs (KO) were identified and annotated. Looking at the top level KEGG BRITE classifications for the pre- and post-cleaning samples (**Supplementary Figure S3**), they all showed a similar distribution. Functions classified as metabolism accounted for the largest group, ranging from 36% to 43%. The next largest group was gene functions involved in genetic information processing, with values between 16% and 21%. Genes not contained in pathway or BRITE also took up a sizeable share, accounting for between 15% and 19%. Cellular processes were slightly less represented, with 11% to 16%, while environmental information processing accounted for an even smaller share with values between 8% and 12%. Genes associated with human diseases, organismal systems, and viral proteins all accounted for less than 1% in every sample.

The FEG9 samples (**Supplementary Figure S4**) showed a very similar distribution to the pre- and post-cleaning samples, with the percentage shares of the different classification groups varying only by a few percent between the sampling campaigns. The distribution also only changed slightly over the course of the sampling period. The biggest difference was that genes involved in cellular processes were slightly more represented than genes not contained in pathway or BRITE.

The most common predicted gene in the pre- and post-cleaning samples was *rpoE*, accounting for an average of 0.338% ± 0.059% of all genes in each sample, followed by *fabG* with an average of 0.269% ± 0.033%. The next most common genes were *ABC.PE.S*, *ABC-2.A*, and *ABC-2.P*, all with approximately 0.25%, and *ABC.PA.S* with 0.241% ± 0.056%. The majority of the highly abundant genes are involved in cellular processes, mostly as transporters or in quorum sensing.

In the FEG9 samples, the most abundant genes were *galE* with an average of 0.314% ± 0.052%, *argE* with 0.313% ± 0.114%, *dgkA* with 0.294% ± 0.087%, and *ASRGL1* with 0.286% ± 0.094%, which are all associated with metabolism. Metabolism-associated genes were much more represented in the most abundant genes in the FEG9 samples compared to pre- and post-cleaning. Genes involved in cellular processes, on the other hand, were less common, with the most abundant of them being *SAM50*, which was only the fifth most abundant gene with 0.250% ± 0.077%.

To gain a better understanding of the pathogenic potential of the greenhouse microbiome, the most abundant human disease-associated genes were identified and calculated which genera were most responsible for their presence. The most abundant disease genes in the pre- and post-cleaning samples (**Supplementary Figure S5**) were *acrB* and *acrA*, which are both involved in beta-lactam resistance. For both of them, *Pseudomonas* was the genus that contributed the most to their abundance, as 22.1% of all *acrB* and 11.4% of all *acrA* hits can be traced back to *Pseudomonas*. Other genera with significant contributions to the presence of these genes were *Stenotrophomonas*, with 4.24% for *acrB* and 4.74% for *acrA*, and *Acinetobacter* with 5.60% for *acrB*, and *Flavobacterium* with 6.20% for *acrA*. *Staphylococcus* showed high contributions to most of the most abundant disease genes, accounting for more than 50% for *ICP*, *rtxA1*, *fhaC*, and *ycfS* and even accounting for 100% of *sspH2* and *ipaH9.8* predictions. Only *oxa*, *SERPINB*, and *adeS* showed no contribution from *Pseudomonas.* Other genera that contributed more than 10% to one or more disease genes were *Stenotrophomonas* for *dsbA* and *adeS*, *Acinetobacter* for rtxA1, *Escherichia-Shigella* for *fimA* and *ycfS*, *Herbaspirillum* for *adeS*, *Ralstonia* for *oxa* and *adeS*, and *Pajaroellobacter* for *SERPINB* and *adeS*. The second-level BRITE classification of these genes shows a relatively even split, with 9 of the 20 most abundant disease genes having a drug resistance function and 11 of them being related to bacterial infections. When looking at the percentage of total human disease-associated genes, drug resistance genes were more abundant, as they accounted for 60.1%.

The most abundant disease genes in the FEG9 samples (**Supplementary Figure S6**) were also *acrB* and *acrA*, with *Pseudomonas* being once again the most significant contributor with 22.5% for *acrB* and 10.2% for *acrA*, which closely matches the values of the pre- and post-cleaning samples. Similar well-matching values could be observed for *Acinetobacter* with a contribution of 7.90% to *acrB* and *Flavobacterium* with a contribution of 11.1% to *acrA*. *Pseudomonas* also contributed significantly to most of the other disease genes, accounting for 50% or more in rxtA1, *fhaC*, *ICP*, *ycfS*, and *fimA* and once more accounting for 100% of sspH2 and ipaH9.8. The only genes it did not contribute to were *pknG*, *aur*, and *blaz*. Other genera with contributions to one or more genes that were higher than 10% include *Stenotrophomonas* for *prtC*, *blaz*, and *aur*, accounting for more than 50% for both of the latter ones, *Acinetobacter* for *rtxA1* and *fimA*, *Variovorax* for *prtC*, *Cupriavidus* for *fhaB* and *prtC*, *Paracoccus* for *prtC*, *Steroidobacter* for *dsbA*, *Klebsiella* for *ycfS*, *Clostridium* for aur, *Haemophilus* for *dsbA*, *Nocardia* and *Corynebacterium* for *pknG*, and *Siccibacter* for *blaZ*. The split between resistance and infection genes was slightly more uneven than in the pre- and post-cleaning samples, with 8 genes associated with drug resistance and 12 associated with bacterial infections. However, when accounting for the abundance of predicted genes, then 54.7% of the human disease-associated genes possess a drug resistance function.

## 4 Discussion

### 4.1 Bioburden

No significant decrease in bioburden could be observed for most sampling positions after cleaning, probably because the MTF was back in operation before the post-cleaning samples were taken. The first seedlings were planted in the MTF 2 weeks before the post-cleaning samples were taken. This gap left ample time for microorganisms to be introduced by the various activities in the MTF and subsequently colonize the cleaned surfaces.

The significant increase in heat shock-resistant organisms in the post-cleaning samples indicates that the cleaning protocol might have failed to eliminate bacterial spores, which are highly resistant to many disinfectants (Russell, 1990). This would have allowed spore formers to repopulate faster, leading to a higher presence in the post-cleaning samples. This hypothesis is also supported by the increased fraction of spore-forming genera in the post-cleaning isolates.

A major factor that might have impacted the cleaning efficacy is the use of a 90%–95% ethanol solution. Surface disinfection is usually carried out with solutions containing between 60% and 70% ethanol (Boyce, 2018), which have been observed to be more effective than close to pure alcohol. A potential explanation for this observation is that the higher water content aids in the penetration of the cell for the ethanol to exhibit its protein-denaturing effects (Yuan et al., 2021). Higher concentrations of ethanol also lead to increased evaporation, lowering the exposure time, potentially to sublethal levels (Rutala and Weber, 2014).

Due to its lack of effectiveness in disinfecting bacterial spores (Thomas, 2012), ethanol disinfection was supplemented with hydrogen peroxide vaporization (HPV) during the cleaning of the MTF. This method is usually effective in deactivating bacterial spores (Klapes and Vesley, 1990), but appeared to have failed in reducing the amount of spore formers in this case. The observed inefficiency might have been caused by potentially lower vapor condensation due to lower humidity in the MTF during the cleaning procedures than during normal operation. Depending on the positioning of the vaporizers, it is also possible that not all surfaces in the MTF were exposed to optimal hydrogen peroxide vapor pressures.

### 4.2 Next-generation sequencing of surface samples

#### 4.2.1 Pre- and post-cleaning

The NGS analysis of the surface samples revealed a multitude of genera that were present in the MTF in significant numbers at various time points. Most of the genera identified in high relative abundance mainly contain environmental species. However, genera like *Pseudomonas*, which consist largely of environmental species (Berlanga, 2010), can also contain species that are potential pathogens. *Pseudomonas syringae* is able to infect the phyllosphere of a wide range of host plants, causing widespread damage (Xin et al., 2018). Other species are able to cause infections in humans. The most prominent one is *Pseudomonas aeruginosa*, which is a major cause of nosocomial infections and often expresses antibiotic resistance, making it hard to treat (Wu et al., 2015). For this reason, it is part of the ESKAPE organisms, made up of *Enterococcus faecium*, *Staphylococcus aureus*, *Klebsiella pneumoniae*, *Acinetobacter baumanii*, *Pseudomonas aeruginosa*, and *Enterobacter* species. The ESKAPE organisms currently pose the largest threat to global health due to their widespread antibiotic resistance, making them dangerous sources of nosocomial infections (Kyriakidis et al., 2021).

*Candidatus Profftella*, which was the most highly abundant genus, has been observed to cause spoilage of ripe grapes in correlation with *Aspergillus* species (Huang et al., 2024), which could imply that it also plays a role in diseases of other plants.

*Acinetobacter* is another genus that was found in high relative abundance. This genus mostly contains environmental species (Doughari et al., 2011). However, the ESKAPE organism *Acinetobacter baumanii* is also a species contained in this genus and thus needs to be monitored closely when identified. *Acinetobacter* species are able to persist in a wide range of environments as well as resist many types of disinfectants (Wisplinghoff et al., 2007). In a clinical setting, the removal of *Acinetobacter* species is carried out through HPV disinfection (Chmielarczyk et al., 2012); thus, the increase in relative abundance of *Acinetobacter* species might be another indication that the HPV protocol for cleaning the MTF was ineffective.

Other identified genera that contain human pathogenic species include *Ralstonia*, with *R. pickettii*, *R. insidosa*, and *R. mannitolilytica* causing osteomyelitis and meningitis in hospital settings (Ryan and Adley, 2014); *Staphylococcus*, which contains another ESKAPE organism with *S. aureus* (Tenover and Gorwitz, 2006); *Cutibacterium*, with *C. acnes* causing implant-associated infections (Gharamti and Kanafani, 2017); and *Moraxella*, with *M. catarrhalis* causing respiratory tract infections (Laura Perez Vidakovics and Riesbeck, 2009).

Another genus containing potential plant pathogens is *Herbaspirillum*, which has also been identified in the FEG sampling sites. Examples include *Herbaspirillum rubrisubalbicans*, which causes symptoms of mottled stripe disease and red stripe disease in crops like sorghum and sugarcane (James et al., 1997; Tan et al., 2010). Some *Herbaspirillum* species have been observed to also cause infections in humans, but only in immunocompromised patients. They can usually be treated well with a range of antibiotics (Bloise et al., 2021).

The alpha diversity analysis of the pre- and post-cleaning samples did not reveal a clear pattern between pre- and post-cleaning. While many sampling positions showed a decrease in the total number of ASVs after cleaning, as indicated by a decrease in the Chao1 index (Kim et al., 2017), others, like FEG9, showed an increased species richness after cleaning. The species diversity weighted toward richness captured by the Shannon index (Fedor and Zvaríková, 2019) was relatively similar for most sampling positions, with minor changes between pre- and post-cleaning. The inverse Simpson index, on the other hand, also captures the species diversity of the samples, but is weighted more toward evenness (Kim et al., 2017), so a more even distribution of species leads to higher values.

Overall, the cleaning did not influence the alpha diversity indices in a consistent way, corroborating the hypothesis that the samples were taken too late after the cleaning, so most effects of the cleaning were already not observable anymore.

When comparing the beta diversity of the pre- and post-cleaning samples, it becomes apparent that most of them remain relatively similar to each other on average. This fits with the observation that no significant cleaning effects were seen, likely due to the time gap between the cleaning and the taking of the post-cleaning samples. The beta diversity analysis also revealed no clear trends as to how the sampling position influenced the diversity.

#### 4.2.2 FEG9 samples

There was some overlap between the most common genera of the pre- and post-cleaning samples and the FEG9 samples, although some specific genera differed significantly in relative abundance. Starting on 28 April 2021, *Citricoccus* species were observed in high relative abundance. The *Citricoccus* genus contains mainly species associated with soils (O’Toole et al., 2023), some of which act as plant growth-promoting bacteria (PGPB) in the rhizosphere (Selvakumar et al., 2015). Because they are often found on and around plant roots, it is likely that the sudden increase in the relative abundance of *Citricoccus* was caused by handling and harvesting plants near the sampling site. For example, a nutrient solution containing rhizosphere bacteria might have dripped from the roots of the plants onto the subfloor when moving the plants between containers. The harvest logs and the surveillance images indicated that the harvest of Asian greens and the transfer of tomato seedlings to their growing trays were the most likely candidates to have introduced this organism at that specific time point.

*Staphylococcus* species were also far more abundant in the FEG9 samples compared to the pre- and post-cleaning samples. *Staphylococcus* species most prominently colonize the skin of humans and other mammals (Kloos, 1980), while a smaller portion is derived from environmental sources like the soil (Nweke and Okpokwasili, 2003). Most species of *Staphylococcus* are commensals, but the genus also contains *S. aureus*, which is another member of the ESKAPE group (Taylor and Unakal, 2023).

The genera *Brevundimonas* and *Nesterenkonia* were also in high relative abundance. They are both associated with various environments, with *Nesterenkonia* species being generally halophilic (Ryan and Pembroke, 2018). They are usually non-threatening to humans and plants. However, *Brevundimonas* species have been observed to cause infections in immunocompromised patients in very rare cases (Han and Andrade, 2005).

The distribution of genera for this period (**Figure 5B**) reveals that the samples were often dominated by a low number of genera. In many cases, these few genera accounted for over 80% of all reads, which explains the low evenness and low richness observed from the alpha diversity measures. A likely cause of this decline in diversity is that environmental stressors such as increased moisture levels on the subfloor because of more condensation, the introduction of new species through plant particles falling onto the subfloor during harvests and the cleaning of the subfloor can all disrupt the established microbial community at the sampling site. This disruption can lead to significant changes in the microbiome composition. Species that are better adapted to these stress factors will be enabled to dominate the community, as they are able to outcompete less well-adapted organisms (Ahmed et al., 2019).

Analyzing the beta diversity of the FEG9 samples reveals that the microbiome composition shifted gradually, but sometimes experienced drastic changes. The sudden shift in microbiome composition can also be seen in the NDMS and the PCoA. However, the Spearman correlation between sampling time and the first component is not significant, while the correlation between sampling time and the second component is significant. This indicates that the gradual changes of the MTF microbiome over time through standard activity cannot explain all the changes, and disruptions, like cleaning of the subfloor, lead to more drastic shifts.

The analysis of the MTF microbiome composition through NGS revealed that it is made up of various environmental organisms. However, some of the identified genera contain species that have the potential to cause harm to the crew members and the plants. This identification approach does not provide a high enough sensitivity to confirm the presence of pathogenic species, but it highlights the importance of monitoring the microbiome composition to identify potential threats. Improvements such as sequencing of the entire 16S rRNA gene instead of only the V1–V2 variable regions might enable a resolution down to the species level (Johnson et al., 2019), which would allow more definite statements about the presence of pathogens. The continuing improvements in sequencing technology will also make it possible to analyze the samples directly at the location where they were taken, instead of having to introduce potential biases through the stresses introduced by transportation and storage.

This will be especially important when BLSS is put to use in space, as regular monitoring of the plant production system microbiome does not seem feasible if the samples have to be sent back to Earth for sequencing. Promising steps in this direction have already been taken by establishing a protocol for real-time microbial profiling onboard the International Space Station using nanopore sequencing (Stahl-Rommel et al., 2021).

### 4.3 Sequencing of isolates

Many of the isolates from the pre- and post-cleaning samples as well as from the FEG9 samples belonged to genera that are part of the *Bacillaceae* family, with *Paenibacillus*, *Bacillus*, and *Fictibacillus* being the most prominent. One thing they all have in common is their ability to form endospores to survive harsh environmental conditions (Nicholson et al., 2000). This causes them to be present in virtually all environments on Earth, so their presence in the samples is unsurprising (Alcaraz et al., 2010). Endospore formation also allows for a much higher survival rate during freezing (Cramm et al., 2019), which could also have increased the fraction of *Bacillaceae* in the colonies grown from the surface swabs.

*Paenibacillus* species are found in various environments like water and soil, but most importantly in the rhizosphere, where various species like *Paenibacillus polymyxa* have been found to act as plant growth-promoting bacteria. Beneficial actions for the host plant include the production of cell-wall-degrading enzymes that target plant pathogenic fungi to protect the host plant (Nielsen and Sørensen, 1997). The production of siderophores also aids the host plant in iron uptake (Sirota-Madi et al., 2010).

Most *Bacillus* species are harmless and can even be beneficial. Some species have useful applications in industry and research (Errington and Aart, 2020), while others act as plant growth-promoting bacteria (Jeong et al., 2012). However, some species are able to cause spoilage in food (Snyder et al., 2024) or serious diseases in humans. The most prominent examples include *Bacillus anthracis*, which causes anthrax (Spencer, 2003), and *Bacillus cereus*, a common cause of food spoilage (Schoeni and Lee Wong, 2005).

*Rossellomorea* species, which were highly abundant in the pre- and post-cleaning samples, are mainly found in marine environments (Bai et al., 2024; Yin et al., 2023). They do not appear to cause any harm to plants or humans, but their ability to oxidize iron has led to them being utilized in groundwater remediation (Lee et al., 2023).

Species of *Chryseobacterium* are found mainly in soil and water, but some species have been isolated from various animals and dairy products (Bernardet et al., 2015). The species has been shown to cause infections in humans in rare cases, mostly in association with immunocompromised patients or indwelling catheters (Mukerji et al., 2016).

While many of the genera identified in the isolates were not found in high abundance levels in the surface samples, there were also some similarities. These include a noticeable abundance of *Staphylococcus* in the pre- and post-cleaning isolates and of *Pseudomonas* in the FEG9 isolates, which were also found in significant numbers in the corresponding surface samples.

The microbiome composition suggested by the sequencing of the isolates, while different from the NGS data, does show a similar picture. Environmental genera dominate the samples, and most of these species can be considered harmless. However, some genera also include species with the potential to cause serious diseases, especially in the cases of *B. cereus* and *B. anthracis*. This highlights the need for continued monitoring of the microbiome composition in the plant growth system to ensure the safety of the crew and the plants.

A direct comparison of the microbiome composition to similar studies is somewhat challenging, as the previous work on the EDEN ISS microbiome only analyzed the composition to the phylum level (Fahrion et al., 2020), and most other microbiome studies in plant growth systems are focused on the plant microbiome. A study on the progressive changes of the rhizosphere in an aeroponics setup identified *Herbaspirillum* and *Methylophilus* among its most abundant genera (Edmonds et al., 2020), which were also among the most abundant genera in the pre- and post-cleaning NGS samples in EDEN ISS. However, there was no overlap between the remaining genera identified in that study and this work. Similar observations could be made when comparing the results with a study focused on the microbiome of the water used for the hydroponic cultivation of tomato plants (Picot et al., 2020). *Mycobacterium* and *Agrobacterium* were found in similar relative abundance levels in both cases, but did not overlap in the other identified genera. A survey on the microbiome of surfaces in a distribution facility handling raw produce (Townsend et al., 2023) could be considered a close analog to this work. In both cases, comparable relative abundance levels of *Pseudomonas* and *Staphylococcus* were observed. Lacking a direct analog, the observed microbiome composition seems to be roughly in line with what has been previously observed for similar environments.

One of the reasons for the small overlap in genera found in the isolates compared to the surface samples might be related to the increased fraction of genera with the potential to form spores in the isolates. The protective properties of the bacterial spore, like the spore coat, as well as its lowered water content compared to a vegetative cell (Cho and Chung, 2020), help the spores survive the freezing process much better than cells unable to form spores. This would lead to a much higher relative abundance of organisms with the potential to form spores in the isolates, as they are able to withstand the stress caused by the freezing much better than vegetative cells and thus should be able to be cultivated from the samples in much higher numbers. However, DNA extraction from bacterial spores is much more challenging compared to extraction from vegetative cells (Mulyukin et al., 2013). The protocol used for DNA extraction in this work was not specialized for extraction from spores, so the actual relative abundance of spore-forming organisms might be even higher than the observed abundance, as spore-derived DNA might have been underrepresented in the samples.

The combined approach of directly sequencing the 16S rRNA gene from surface samples as well as the cultivated isolates provides multiple benefits. 16S rRNA gene amplicon sequencing provides a good overview of the composition of the studied microbiome, but the selection of parameters like the primer pairs, databases, and bioinformatic settings can have a significant impact on the taxa detected and can lead to certain taxa being underrepresented or even missed completely (Abellan-Schneyder et al., 2021). While advancements in sequencing technology, like long-read sequencing, can reduce amplification bias by sequencing the entire 16S rRNA gene (Johnson et al., 2019), the choice of the clustering method and database still needs to be considered carefully. 16S rRNA gene amplicon sequencing also does not provide any information on whether or not the reads were derived from viable cells. The cultivation-based approach, on the other hand, does ensure that all identified isolates originate from living cells cultivated from the sample. The major drawback of this method is that only a small fraction of the organisms present in the sampled environment can be cultivated with the current cultivation methods (Pedrós-Alió and Manrubia, 2016). The fraction captured in this study is likely to be even lower, as only a single culture condition (R2A agar with cycloheximide, room temperature, oxic conditions) was used. Nonetheless, cultivation-based approaches should not be disregarded, as they can reveal the presence of taxa missed by the sequencing-based approaches. This can be especially advantageous if species or genera of critical importance for the current research question are found, as they can be further characterized with culture-based analysis approaches. Evidence that the culture-dependent approach was helpful in this study is that many isolates were characterized as genera like *Bacillus*, *Paenibacillus*, and *Fictibacillus*, which were not found in the surface samples in significant numbers. These results highlight the importance of combining cultivation- and sequencing-based approaches to gain a more complete image of the actual microbiome composition.

### 4.4 Functional analysis of the greenhouse microbiome

The lack of studies on the surface microbiomes of greenhouses makes the comparison of the functional profile of the FEG with other greenhouses difficult. However, when comparing the percentages of the major KEGG BRITE categories to the main categories in the *Clostridium difficile* core genome (Kulecka et al., 2021), they appear to match relatively well, with the largest category in *C. difficile* also being metabolism, with 30.5% of all reads, followed by information processing with 20.7% and cellular processes with 9.3%. All of these values are approximately 5%–10% lower than what was observed for the EDEN ISS microbiome, but the order of the categories is the same between both studies.

The fact that *Pseudomonas* accounts for the largest number of predicted disease genes might imply that pathogenic species like *P. aeruginosa* were present. However, none of the genes encoding for its exotoxins *ExoS*, *ExoT*, *ExoU*, and *ExoY* secreted by type III secretion systems (Hauser, 2009) or for exotoxin A that is secreted by type II secretion systems (Pugsley et al., 1990) were predicted. Of the genes encoding for its secreted proteases important for invasion (Bielecki et al., 2008), only *LasB* was predicted, but with very few hits and only in the pre- and post-cleaning samples. The lack of these important virulence factors could indicate that *P. aeruginosa* was not present, but the predicted presence of the *ipaH9.8* and *sspH2* genes, which are both E3 ubiquitin ligases that are injected into host cells (Bhavsar et al., 2013; Keszei and Sicheri, 2017), still points toward some potential pathogenic function. *Stenotrophomonas* is a genus of bacteria distributed widely in the environment, but found most often in association with plants as PGPB (Ryan et al., 2009), which makes their presence in the FEG unsurprising. Their contribution to the predicted disease-related genes is due to their wide range of antimicrobial resistances, which has been especially observed for *Stenotrophomonas maltophilia* (Minkwitz and Berg, 2001). *Stenotrophomonas maltophilia* can also act as an opportunistic pathogen, in which case the antimicrobial resistance can make effective treatment of the infection challenging (Trifonova and Strateva, 2019). Several of the *S. maltophilia* antimicrobial resistance genes like the *smeABC* operon (Liao et al., 2021), the *floR* gene (Toleman et al., 2007), or the *dfrA1* and *dfrA12* genes (Hu et al., 2011) were predicted, but genes for important virulence factors like the serin proteases StmPr1-3 (Bhaumik et al., 2023) or the effector molecules TcfA and TcfB (Nas et al., 2021) were not predicted. *Stenotrophomonas* showed a strong contribution toward the prediction of *aur*, the aureolysin gene of *S. aureus* (Sabat et al., 2000), which is a metalloproteinase belonging to a protein family that encompasses virulence factors for a range of pathogens. While this indicates that the presence of pathogenic *S. maltophilia* strains is possible, it appears less likely due to the lack of other virulence factor genes. The prediction of virulence factor genes also does not necessarily mean that they are expressed. Furthermore, a majority of the human disease-associated genes that were predicted encode for antimicrobial resistance genes instead of virulence factors, which does not necessarily imply a pathogenic function (Dionisio et al., 2023). While the presence of pathogenic species can neither be confirmed nor denied from this information, it nonetheless points toward them not being present in significant numbers. Other genera that had been identified through the 16S rRNA sequencing and contained potentially pathogenic species, like *Acinetobacter* or *Herbaspirillum*, were also among the major contributors toward the common disease genes, but were also lacking predictions for important virulence factors. Overall, the results of the functional predictions indicate that pathogenic species were not likely to be present in high abundance in the FEG, but the not insignificant number of predicted disease-associated genes once again highlights the need for a comprehensive microbial monitoring. In conclusion, this work provides a strong case for microbial monitoring in BLSS, as multiple genera that contain potentially harmful species, including some ESKAPE organisms, were identified. The lack of a clear reduction in bioburden also revealed shortcomings in the currently employed cleaning regimen. Improvements like using a more suitable ethanol concentration for disinfection and improved settings for the HPV disinfection for spore removal are necessary to reduce the number of potential pathogens. This work also clearly showed the discrepancies between culture-dependent and culture-independent approaches for microbiome analysis, with multiple genera found in high abundance through one method not being identified by the other, highlighting the advantages of a combined approach to obtain a more complete picture of the microbiome composition. The methods used in this work could be supplemented in the future by DNA extraction protocols more suited to bacterial spores and include the sequencing of fungal DNA to capture a wider range of potentially pathogenic organisms.

## Data Availability

The datasets presented in this study can be found in online repositories. The names of the repository/repositories and accession number(s) can be found below: https://figshare.com/, https://doi.org/10.6084/m9.figshare.28731461.v1 https://figshare.com/, https://doi.org/10.6084/m9.figshare.28731509.v1 https://figshare.com/, https://doi.org/10.6084/m9.figshare.28732151.v1 https://figshare.com/, https://doi.org/10.6084/m9.figshare.28732199.v1 https://figshare.com/, https://doi.org/10.6084/m9.figshare.28737851.v1 https://figshare.com/, https://doi.org/10.6084/m9.figshare.28737857.v1 https://figshare.com/, https://doi.org/10.6084/m9.figshare.28737926.v1 https://figshare.com/, https://doi.org/10.6084/m9.figshare.28737935.v1.
