# Supplementary material for: The EDEN ISS mobile test facility microbiome changes by cleaning and continued use
Source: Front Microbiomes. 2025 Oct 17;4:1608732. doi: 10.3389/frmbi.2025.1608732 (PMC12993677; doi:10.3389/frmbi.2025.1608732)
Supplement: Supplementary file 1 [file DataSheet1.docx]

**Supplementary Material**

**Supplementary table 1: pairwise PERMANOVA of the pre- and post-cleaning samples**

Pairwise PERMANOVA was performed with the pre- and post-cleaning samples, comparing both the sampling locations and between pre- and post-cleaning. Pairings with significant differences (p < 0.05) are highlighted in red.

| **Pairwise PERMANOVA: sampling location** | | | | |
| --- | --- | --- | --- | --- |
| **pairs** | **SumsOfSqs** | **F.Model** | **R^2^** | **p-value** |
| FEG01 vs FEG02 | 0.058 | 4.033 | 0.402 | 0.020 |
| FEG01 vs FEG03 | 0.042 | 2.918 | 0.327 | 0.137 |
| FEG01 vs FEG04 | 0.009 | 0.448 | 0.070 | 0.810 |
| FEG01 vs FEG05 | 0.030 | 1.474 | 0.197 | 0.209 |
| FEG01 vs FEG06 | 0.009 | 0.7164 | 0.107 | 0.460 |
| FEG01 vs FEG07 | 0.074 | 5.777 | 0.491 | 0.031 |
| FEG01 vs FEG08 | 0.025 | 2.529 | 0.297 | 0.271 |
| FEG01 vs FEG09 | 0.004 | 0.235 | 0.038 | 0.719 |
| FEG01 vs FEG10 | 0.044 | 4.273 | 0.416 | 0.031 |
| FEG02 vs FEG03 | 0.0201 | 1.920 | 0.242 | 0.200 |
| FEG02 vs FEG04 | 0.034 | 2.190 | 0.2679 | 0.150 |
| FEG02 vs FEG05 | 0.007 | 0.431 | 0.067 | 0.630 |
| FEG02 vs FEG06 | 0.065 | 7.222 | 0.546 | 0.032 |
| FEG02 vs FEG07 | 0.006 | 0.669 | 0.100 | 0.518 |
| FEG02 vs FEG08 | 0.102 | 16.4542 | 0.732 | 0.026 |
| FEG02 vs FEG09 | 0.056 | 4.578 | 0.433 | 0.064 |
| FEG02 vs FEG10 | 0.026 | 4.055 | 0.403 | 0.035 |
| FEG03 vs FEG04 | 0.017 | 1.076 | 0.152 | 0.400 |
| FEG03 vs FEG05 | 0.017 | 1.070 | 0.151 | 0.273 |
| FEG03 vs FEG06 | 0.032 | 3.561 | 0.372 | 0.104 |
| FEG03 vs FEG07 | 0.016 | 1.811 | 0.232 | 0.202 |
| FEG03 vs FEG08 | 0.103 | 17.070 | 0.740 | 0.031 |
| FEG03 vs FEG09 | 0.0368 | 3.022 | 0.335 | 0.062 |
| FEG03 vs FEG10 | 0.015 | 2.409 | 0.286 | 0.108 |
| FEG04 vs FEG05 | 0.015 | 0.686 | 0.103 | 0.470 |
| FEG04 vs FEG06 | 0.009 | 0.608 | 0.092 | 0.681 |
| FEG04 vs FEG07 | 0.041 | 2.962 | 0.331 | 0.045 |
| FEG04 vs FEG08 | 0.047 | 4.228 | 0.413 | 0.056 |
| FEG04 vs FEG09 | 0.008 | 0.442 | 0.069 | 0.644 |
| FEG04 vs FEG10 | 0.025 | 2.225 | 0.271 | 0.058 |
| FEG05 vs FEG06 | 0.040 | 2.707 | 0.311 | 0.097 |
| FEG05 vs FEG07 | 0.015 | 1.023 | 0.146 | 0.381 |
| FEG05 vs FEG08 | 0.059 | 5.012 | 0.4555 | 0.029 |
| FEG05 vs FEG09 | 0.028 | 1.544 | 0.205 | 0.230 |
| FEG05 vs FEG10 | 0.024 | 1.960 | 0.2461 | 0.143 |
| FEG06 vs FEG07 | 0.078 | 10.304 | 0.632 | 0.032 |
| FEG06 vs FEG08 | 0.055 | 11.743 | 0.662 | 0.030 |
| FEG06 vs FEG09 | 0.009 | 0.792 | 0.117 | 0.499 |
| FEG06 vs FEG10 | 0.036 | 7.274 | 0.548 | 0.031 |
| FEG07 vs FEG08 | 0.130 | 27.924 | 0.823 | 0.024 |
| FEG07 vs FEG09 | 0.072 | 6.646 | 0.526 | 0.035 |
| FEG07 vs FEG10 | 0.0342 | 6.907 | 0.535 | 0.036 |
| FEG08 vs FEG09 | 0.026 | 3.266 | 0.352 | 0.079 |
| FEG08 vs FEG10 | 0.095 | 45.432 | 0.8831 | 0.030 |
| FEG09 vs FEG10 | 0.036 | 4.397 | 0.423 | 0.064 |
| **Pairwise PERMANOVA: pre- and post-cleaning** | | | | |
| **pairs** | **SumsOfSqs** | **F.Model** | **R^2^** | **p-value** |
| pre-cleaning vs post-cleaning | 0.032 | 1.843 | 0.046 | 0.142 |

**Supplementary Table 2: Denoising stats**

| **Denoising stat** | **First sequencing run** | **Second sequencing run** |
| --- | --- | --- |
| Reads before truncating | 157056 | 100447 |
| Reads after truncating | 14468 | 86255 |
| Denoised forward reads | 13498 | 84220 |
| Denoised reverse reads | 133927 | 84334 |
| Merged sequences | 115240 | 77395 |
| Non-chimeric sequences | 91702 | 46748 |
| Decontam thresholds | 0.1 | 0.4 |
| Reads after removing mitochondria and chloroplast reads and filtering with decontam | 90545 | 44938 |
| Archaeal reads | 0 | 0 |

**Supplementary Figure 1: Principle Component analysis (PCoA) of the Pre- and post-cleaning samples**

The PCoA of the pre- and post-cleaning samples was calculated with the Bray-Curtis dissimilarity and plotted for the first and second principal component. The data points were colored according to the sampling location and the shape was determined by the sampling time.

**Supplementary Figure 2: Principle Component analysis (PCoA) of the FEG9 samples**

The PCoA of the FEG9 samples was calculated with the Bray-Curtis dissimilarity and plotted for the first and second principal component. The data points were colored according to the sampling location and the shape was determined by the sampling time.

**Supplementary Figure 3: KEGG BRITE categories of the genes predicted from the Pre- and post-cleaning samples**

The genes predicted from the 16S rRNA sequences with Picrust2 were annotated with the KEGG BRITE database and the percentage share of each top level category for each pre- and post-cleaning sample was plotted.

**Supplementary Figure 4: KEGG BRITE categories of the genes predicted from the FEG9 samples**

The genes predicted from the 16S rRNA sequences with Picrust2 were annotated with the KEGG BRITE database and the percentage share of each top level category for each FEG9 sample was plotted.

**Supplementary Figure 5: Genera accounting for disease genes – Pre- and post-cleaning**

The 20 genera that led to the most hits for the prediction of human disease associated genes were identified. The number of hits was then normalized to their abundance in the pre- and post-cleaning samples and the percentage that each of those genera accounted for each of the 20 most common disease genes was calculated and plotted.

**Supplementary Figure 6: Genera accounting for disease genes – FEG9**

The 20 genera that led to the most hits for the prediction of human disease associated genes were identified. The number of hits was then normalized to their abundance in the FEG9 samples and the percentage that each of those genera accounted for each of the 20 most common disease genes was calculated and plotted.
