# Supplementary figures and images for: The EDEN ISS mobile test facility microbiome changes by cleaning and continued use
Source: Front Microbiomes. 2025 Oct 17;4:1608732. doi: 10.3389/frmbi.2025.1608732 (PMC12993677; doi:10.3389/frmbi.2025.1608732)

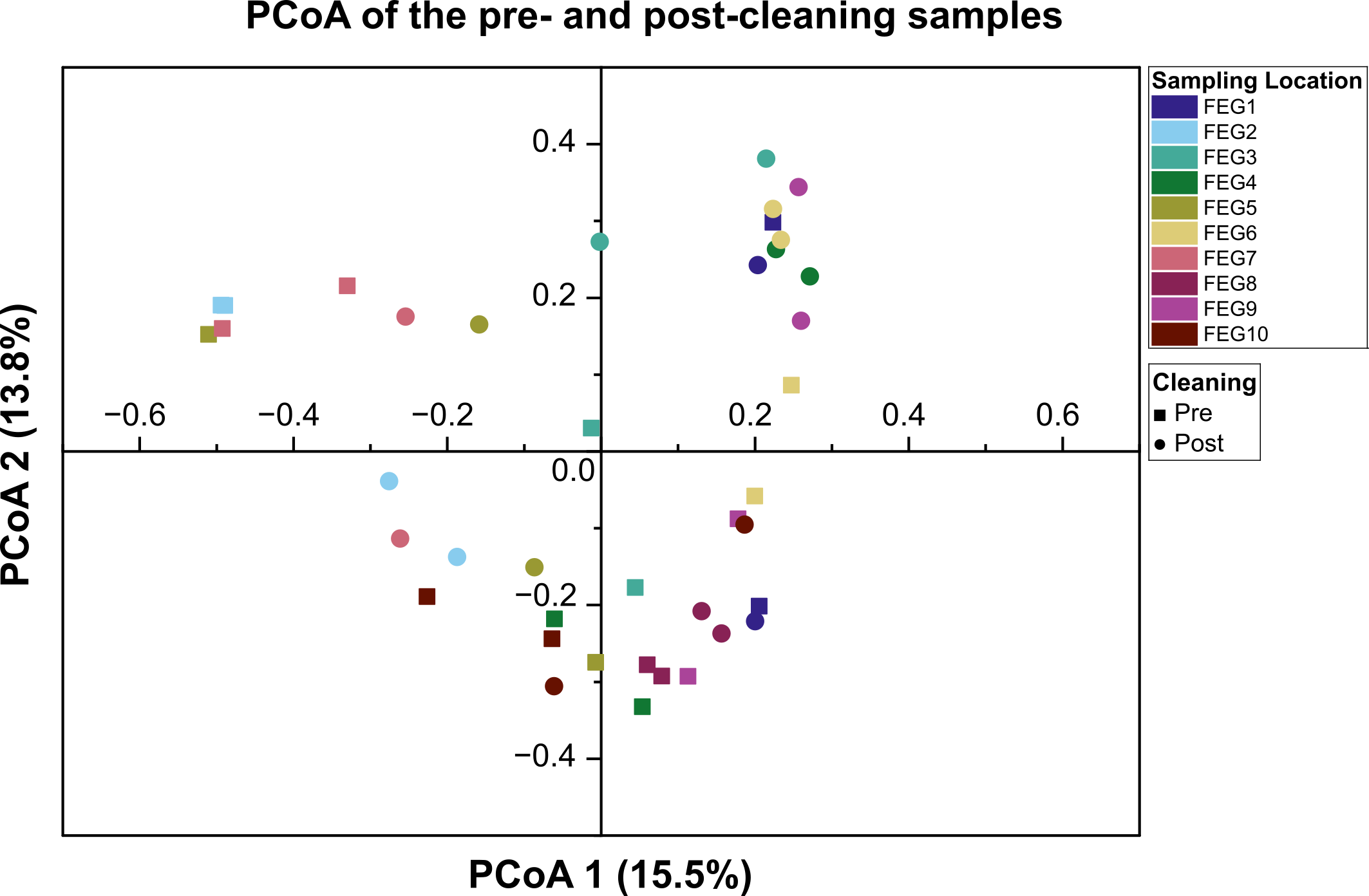

Supplement: Supplementary file 2 [file Image1.tiff]

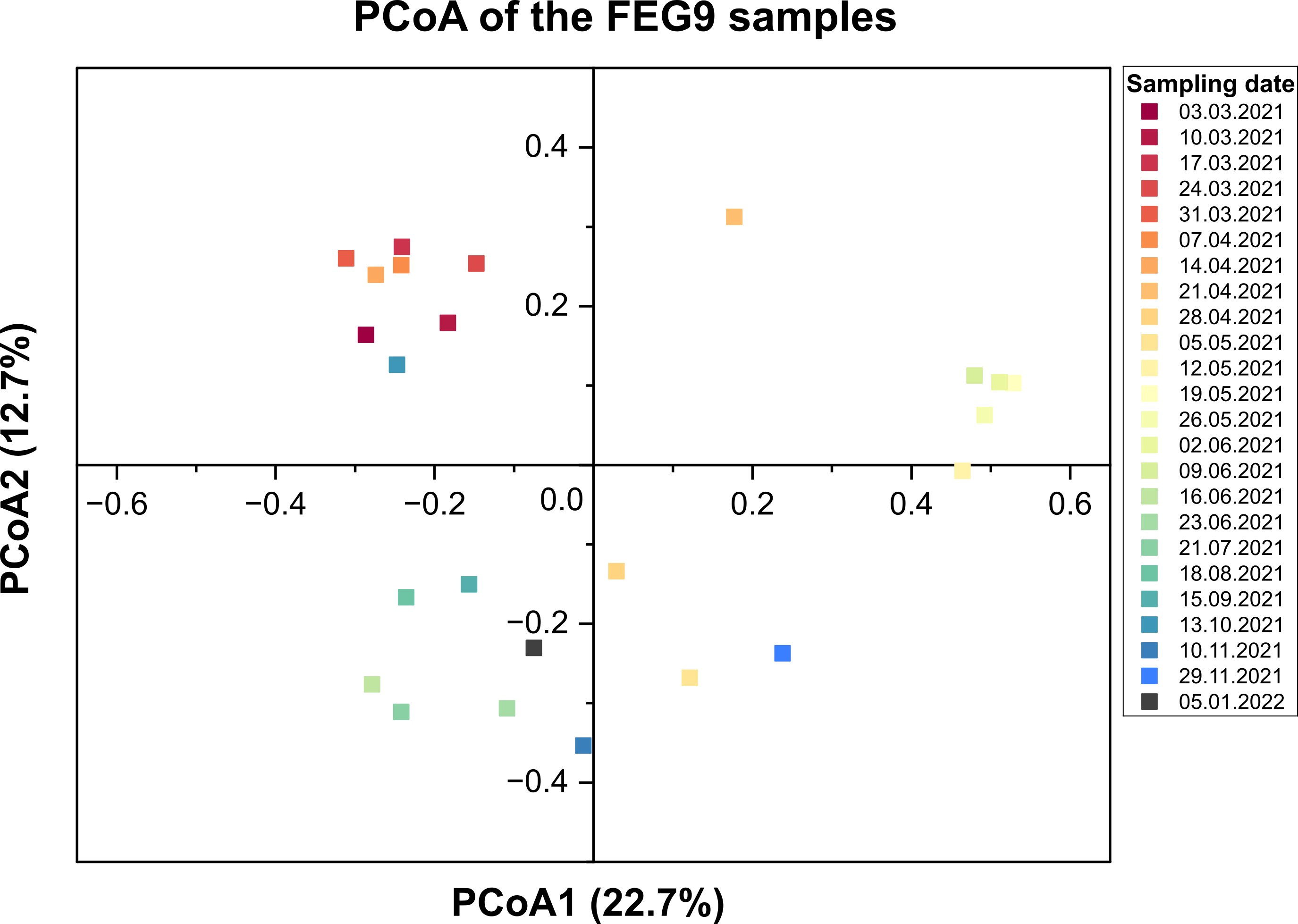

Supplement: Supplementary file 3 [file Image2.tiff]

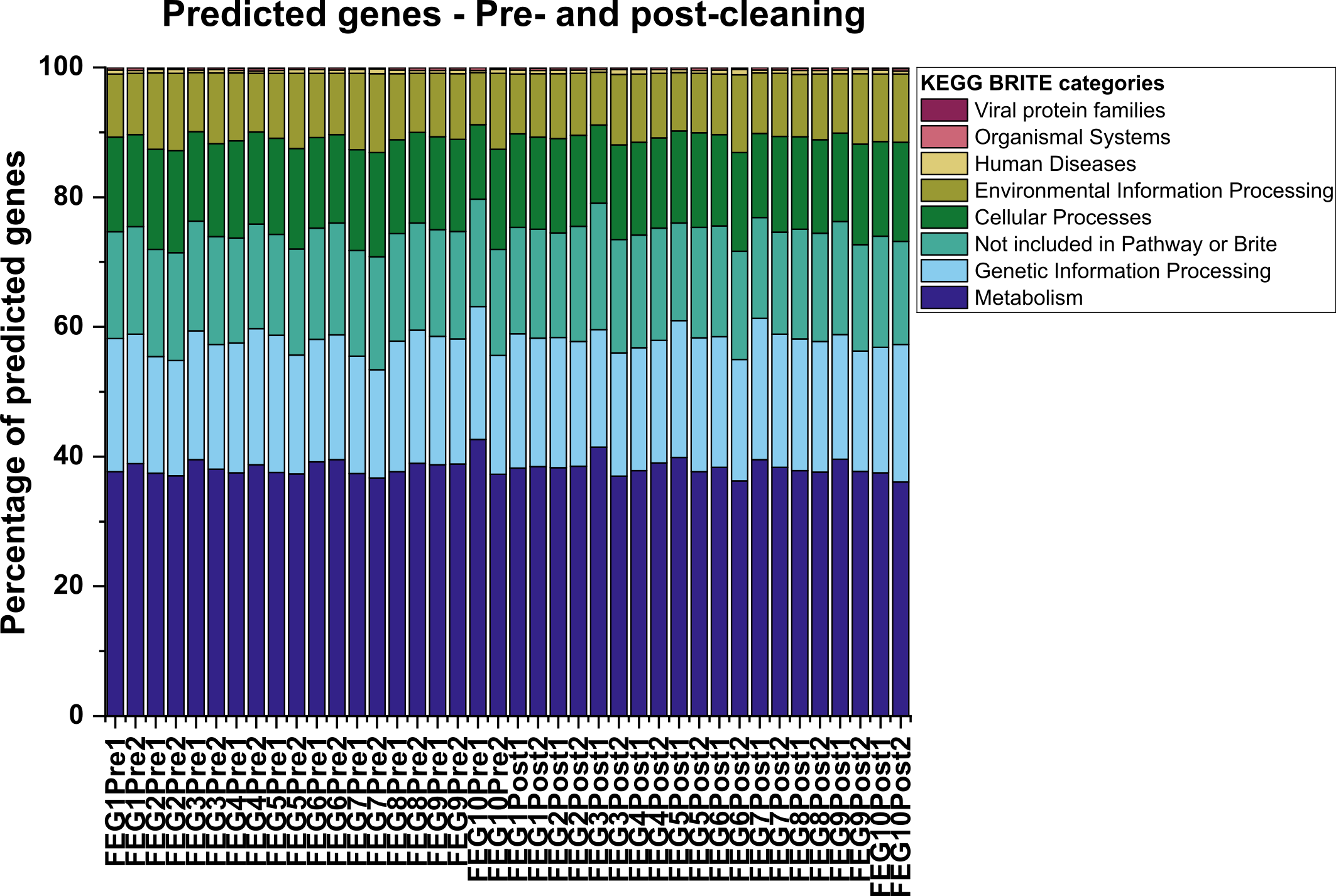

Supplement: Supplementary file 4 [file Image3.tiff]

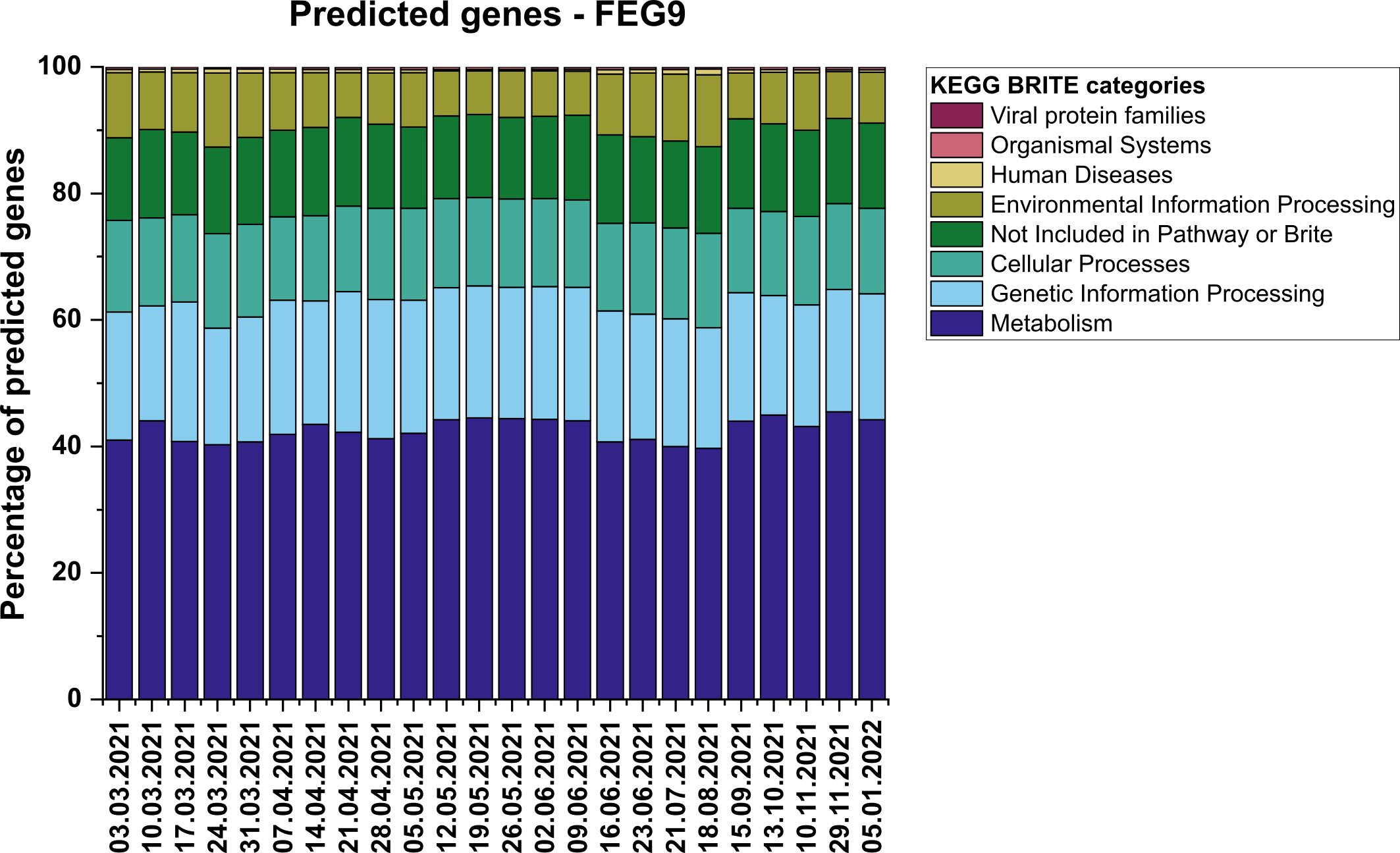

Supplement: Supplementary file 5 [file Image4.tiff]

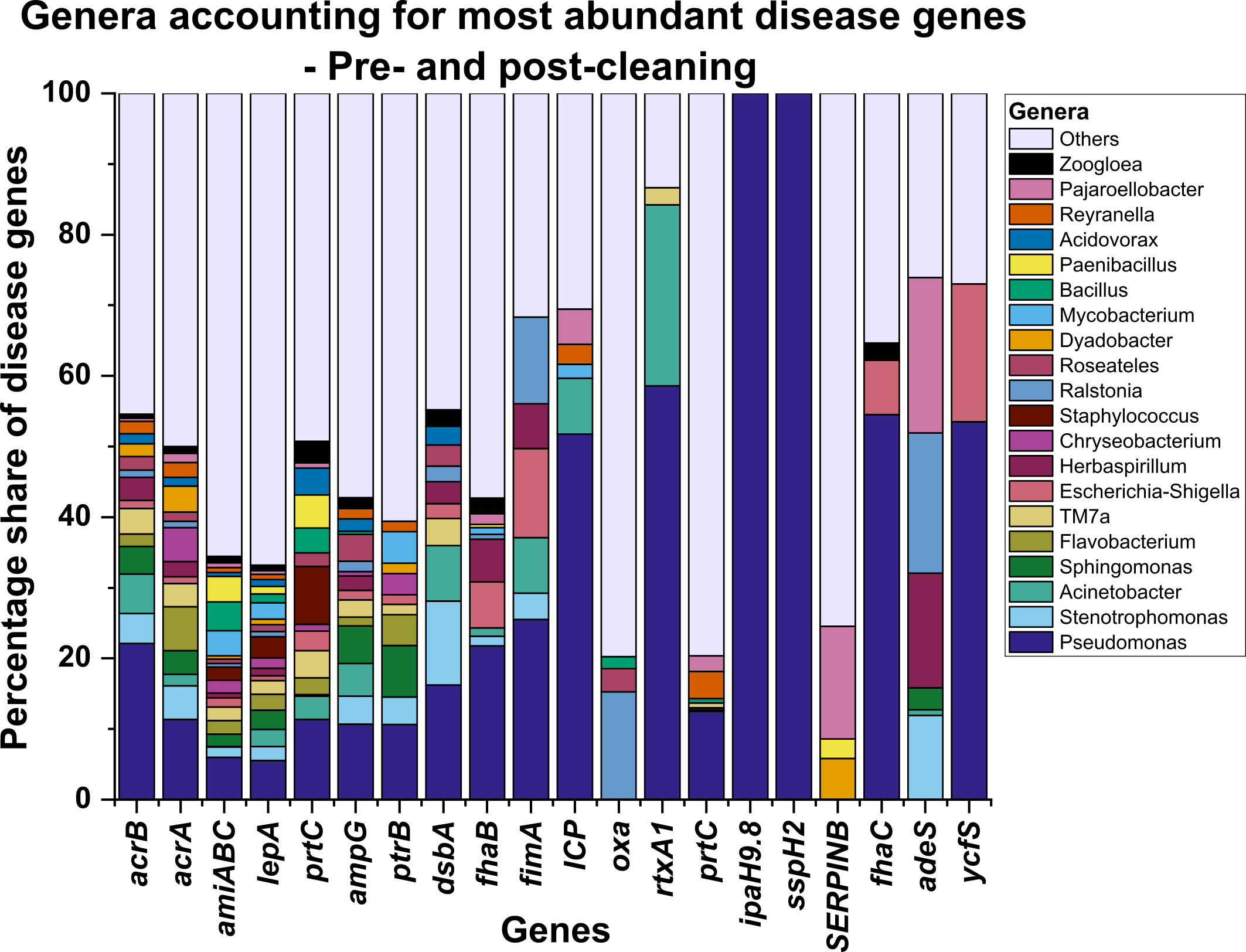

Supplement: Supplementary file 6 [file Image5.tiff]

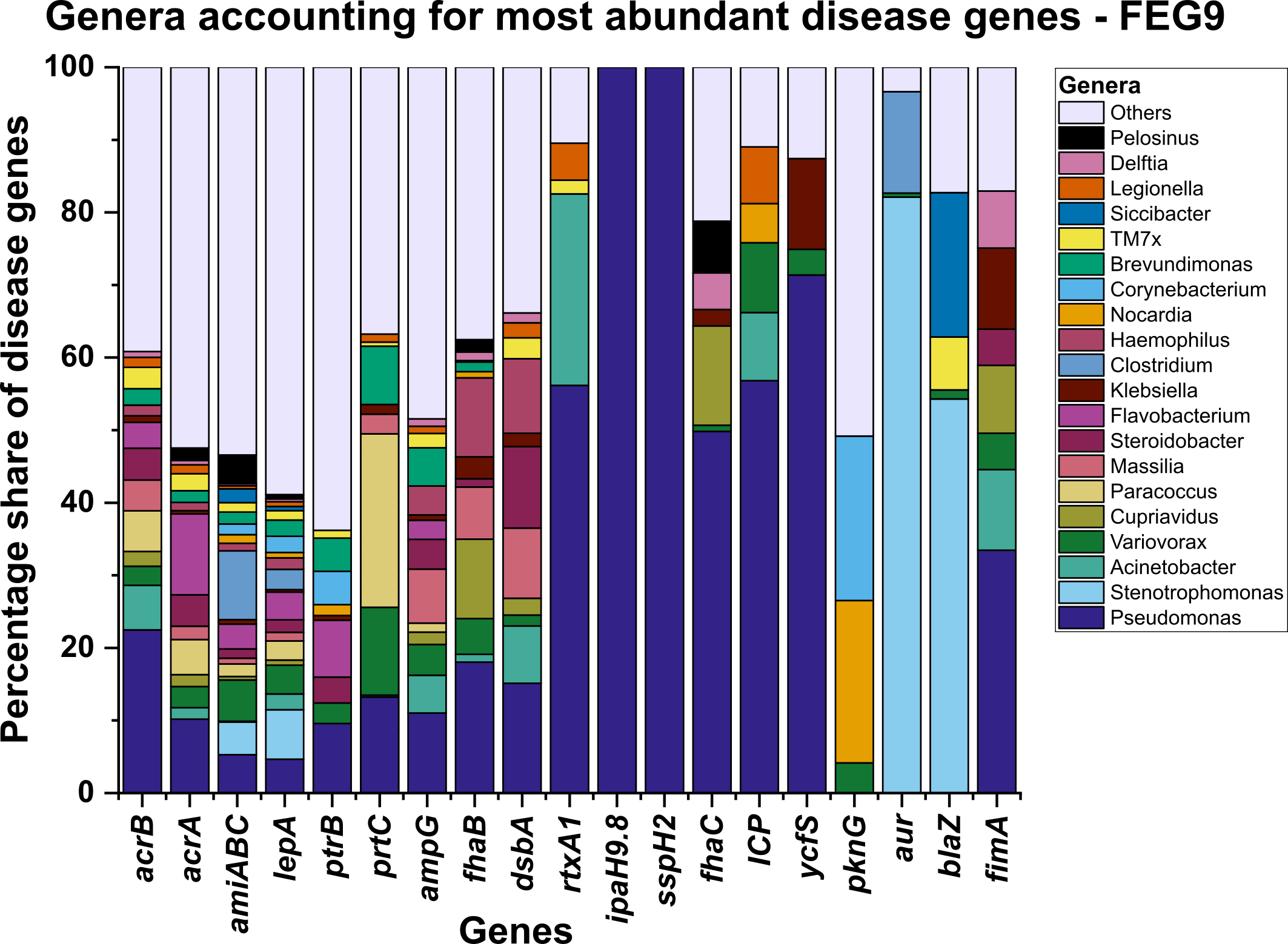

Supplement: Supplementary file 7 [file Image6.tiff]
